# Supplementary material for: Discovery of a Human Metabolite That Mimics the Bacterial Quorum-Sensing Autoinducer AI‑2
Source: J Am Chem Soc. 2026 Feb 4;148(6):6258–69. doi: 10.1021/jacs.5c18527 (PMC12921845; doi:10.1021/jacs.5c18527)
Supplement: Supplementary file 1 [file ja5c18527_si_001.pdf]

Supporting Information

Discovery of a Human Metabolite that Mimics the Bacterial Quorum-Sensing Autoinducer AI-2

Emilee E. Shine[1], Julie S. Valastyan[1,2], Vanessa Y. Ying[3], Jonathan Z. Huang[3], Mohammad R. Seyedsayamdost[1,3]*, and Bonnie L. Bassler[1,2]*

*corresponding authors

1. Department of Molecular Biology, Princeton University, Princeton, New Jersey 08544, United States.

2. Howard Hughes Medical Institute, Chevy Chase, Maryland 20815, United States.

3. Department of Chemistry, Princeton University, Princeton, New Jersey 08544, USA

Table of Contents

Figures S1 to S17……………………………………………………………………..S2

Tables S1 to S3 ………………………………………………………………………S17

Synthetic methods and characterization data for all synthetic compounds…………………..S19

Supplementary References…………………………………………………………...S43

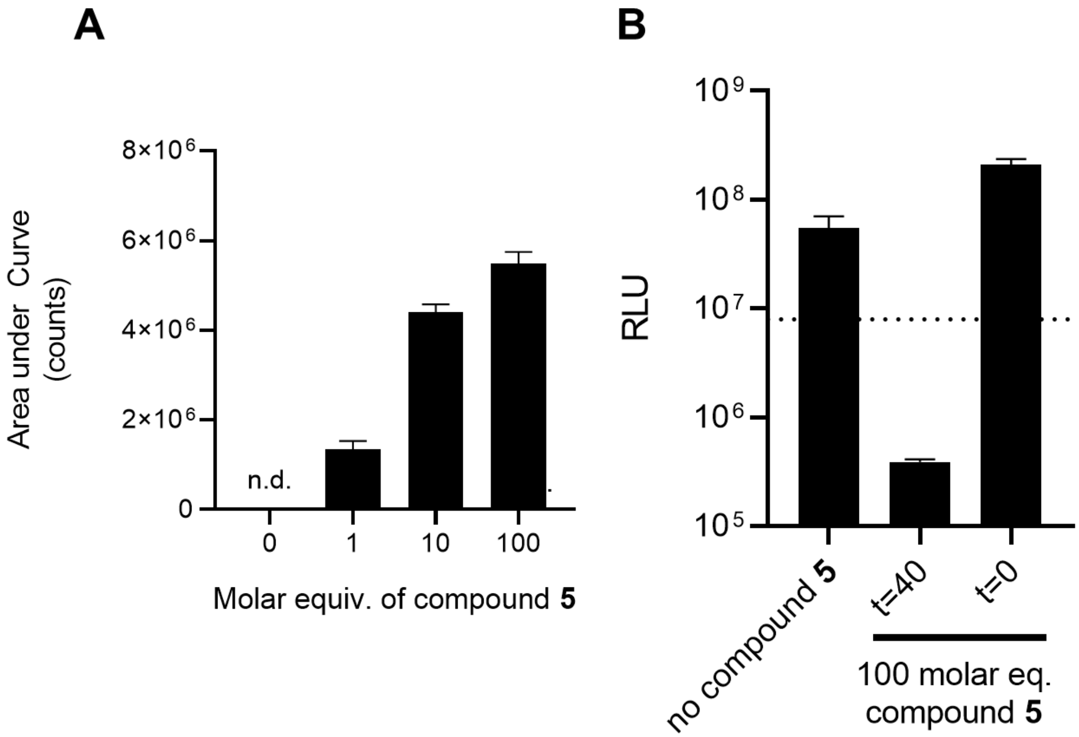

Figure S1. Optimization of DPD (**1**) derivatization with DMB (**5**). A) Relative quantitation of DPD-quinoxaline (**6**) measured by LC-MS. In each reaction, 5 nmol **1** was incubated with the indicated molar equivalents of **5**. Reactions were carried out at 55°C for 40 min. Data were quantified as area under the peaks for extracted *m/z* 249.1. Error bars denote standard deviations for biological replicates, n = 3. B) Bioluminescence output from the *V. harveyi* TL-26 bioassay strain. In each reaction, 5 nmol **1** was incubated alone or with 100 molar equivalents of **5**. t = 0 indicates that compound **5** and compound **1** were added simultaneously to the reporter strain without prior reaction. t = 40 min indicates that compound **5** and compound **1** were reacted at 55°C for 40 min before exposure to the reporter strain. In B, RLU denotes relative light units, which are bioluminescence/OD_{600}. n.d. = not detected.

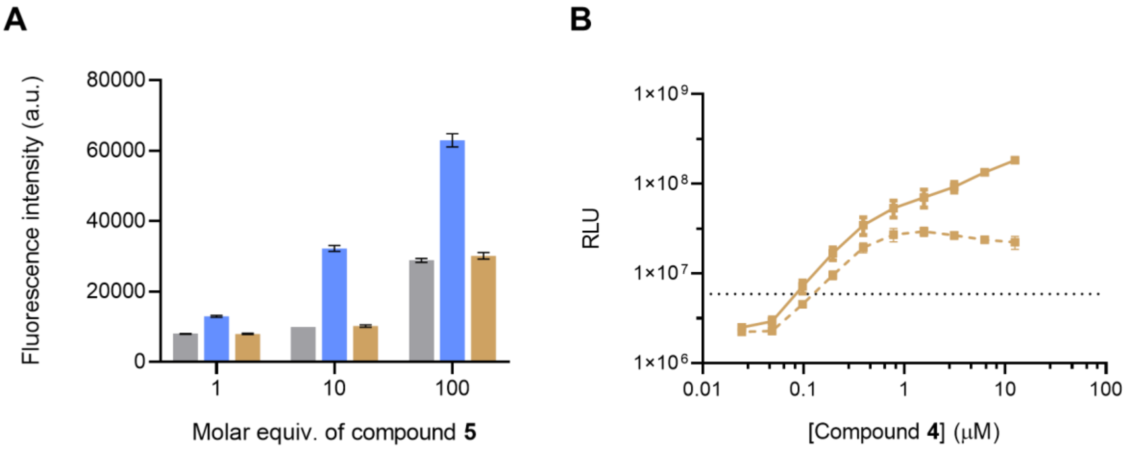

Figure S2. MHF, **4**, which lacks an a-diketone, is not derivatized by DMB, **5**. A) Fluorescence intensities of reaction mixtures containing water (gray), 10 nmol of **1** (blue) or 100 nmol of **4** (gold) incubated with the indicated molar equivalents of **5**. Production of a fluorescent quinoxaline product was detected at excitation/emission 355 nm/393 nm. B) Reactions containing 500 µM of **4** were incubated with either control solution (solid line) or 3 mM **5** (dashed line) for 40 min at 55°C followed by application in serial dilution to the *V. harveyi* TL-26 bioassay strain. RLU denotes relative light units, which are bioluminescence/OD_{600}, and the dotted line indicates the baseline light output in the bioassay. In both panels, error bars denote standard deviations for biological replicates, *n* = 3.

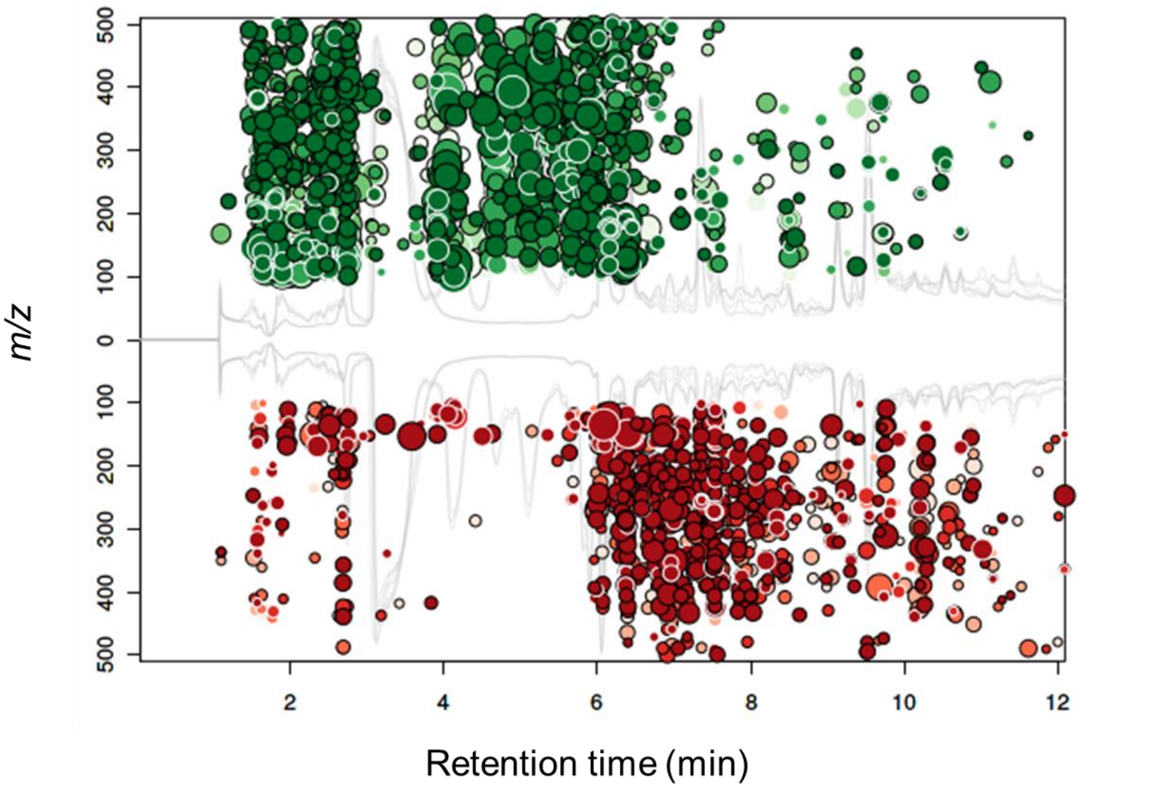

Figure S3. Metabolome cloud plot generated from XC-MS Online comparing mammalian AI-2 mimic samples derivatized with **5** versus PBS treated with **5**. Each point represents a distinct molecular feature, with point sizes proportional to fold-changes and colors corresponding to *p*-values (n = 3 biological replicates). Darker colors indicate lower *p*-values. Retention times are represented by positions on the x-axis. Mass-to-charge ratios (*m/z*) are represented by positions on the y-axis. Points in green represent molecular features enriched in the AI-2 mimic sample treated with compound **5**. Points in red represent molecular features enriched in the control.

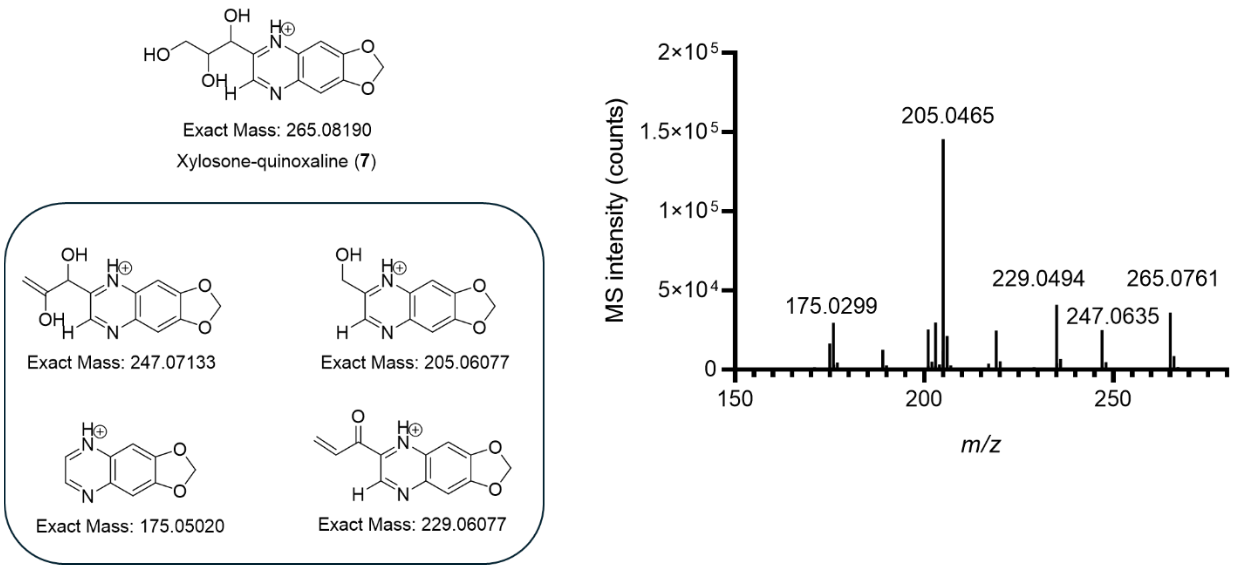

Figure S4. MS/MS spectrum of **7** resulting from synthesized **8** derivatized with **5**. Shown in the box are predicted fragment ion structures are shown with computed exact masses calculated for [M+H]. Observed *m/z* fragments are labeled in the spectrum.

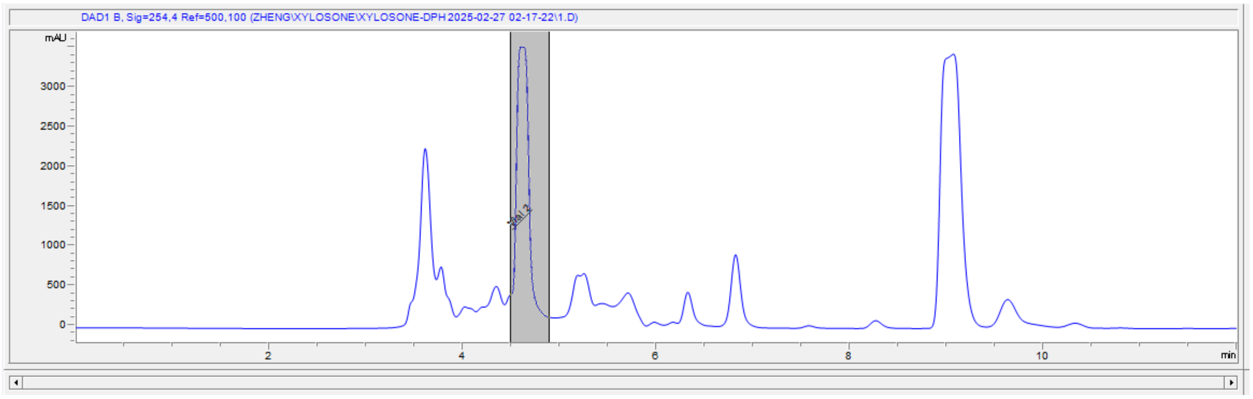

Figure S5. HPLC trace for the purification of L-xylosone DPH derivative.

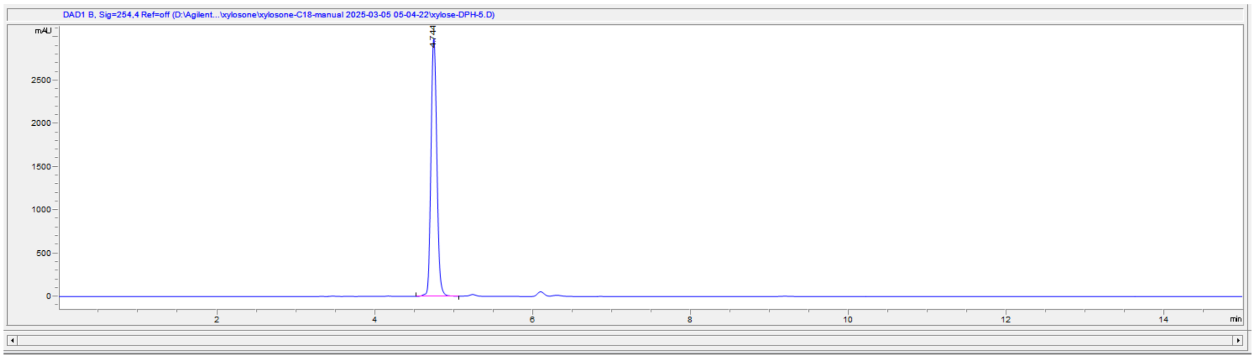

File Information	
LC-File	xylose-DPH-5.D
File Path	D:\Agilent\Zheng\xylosone\xylosone-C18-manual 2025-03-05 05-0
Date	05-Mar-25, 05:38:19
Sample	xylose-DPH-5
Sample Info	
Barcode	
Operator	SYSTEM
Method	xylosone.M
Reference	
Analysis Time	14.993 min
Sampling Rate	0.0067 min (0.402 sec), 2250 datapoints

#	Time	Type	Area	Height	Width	Area%	Symmetry
1	4.744	BB	15393	2985.2	0.0802	100.000	0.864

Figure S6. Representative HPLC trace for the 5 mg/mL stock of L-xylosone DPH derivative.

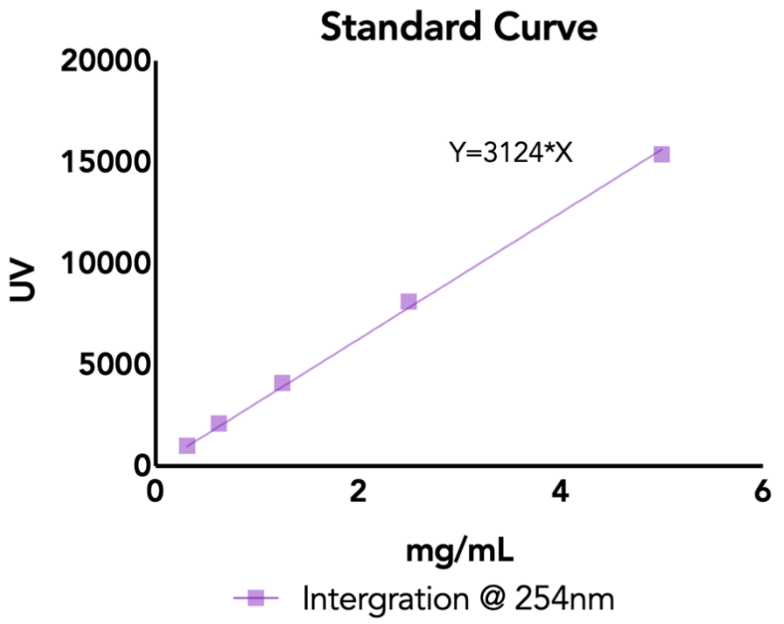

Figure S7. Standard curve for the HPLC-UV quantification of DPH-modified xylosone.

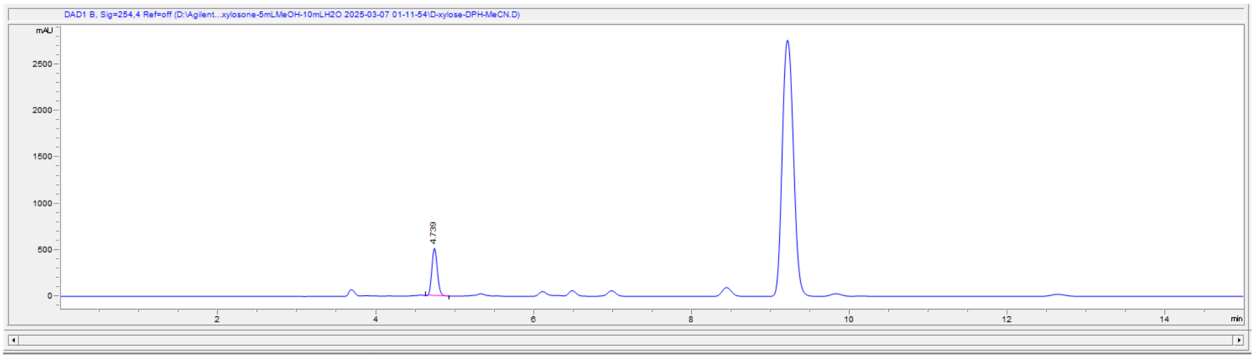

File Information	
LC-File	D-xylose-DPH-MeCN.D
File Path	D:\Agilent\Zheng\xylosone\xylosone-5mLMeOH-10mLH2O 2025-03-
Date	07-Mar-25, 01:46:05
Sample	D-xylose-DPH-MeCN
Sample Info	
Barcode	
Operator	SYSTEM
Method	xylosone.M
Reference	
Analysis Time	14.993 min
Sampling Rate	0.0067 min (0.402 sec), 2250 datapoints

#	Time	Type	Area	Height	Width	Area%	Symmetry
1	4.739	BB	2507.7	514.3	0.0769	100.000	0.864

Figure S8. HPLC trace for DPH-modified D-xylosone. The concentration of the initial D-xylosone stock is calculated to be 6.4 mM.

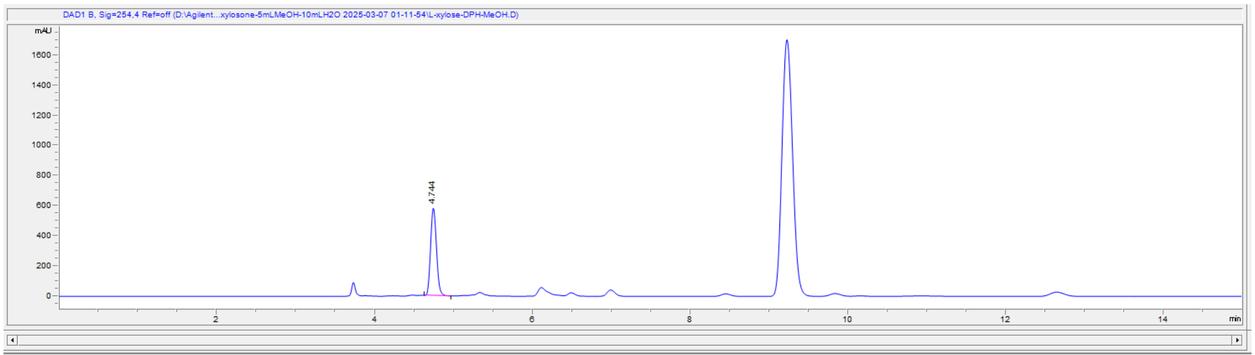

File Information	
LC-File	L-xylose-DPH-MeOH.D
File Path	D:\Agilent\Zheng\xylosone\xylosone-5mLMeOH-10mLH2O 2025-03-
Date	07-Mar-25, 01:29:42
Sample	L-xylose-DPH-MeOH
Sample Info	
Barcode	
Operator	SYSTEM
Method	xylosone.M
Reference	
Analysis Time	14.993 min
Sampling Rate	0.0067 min (0.402 sec), 2250 datapoints

#	Time	Type	Area	Height	Width	Area%	Symmetry
1	4.744	BB	3024.2	582	0.0806	100.000	0.839

Figure S9. HPLC trace for DPH-modified L-xylosone. The concentration of the initial L-xylosone stock is calculated to be 7.7 mM.

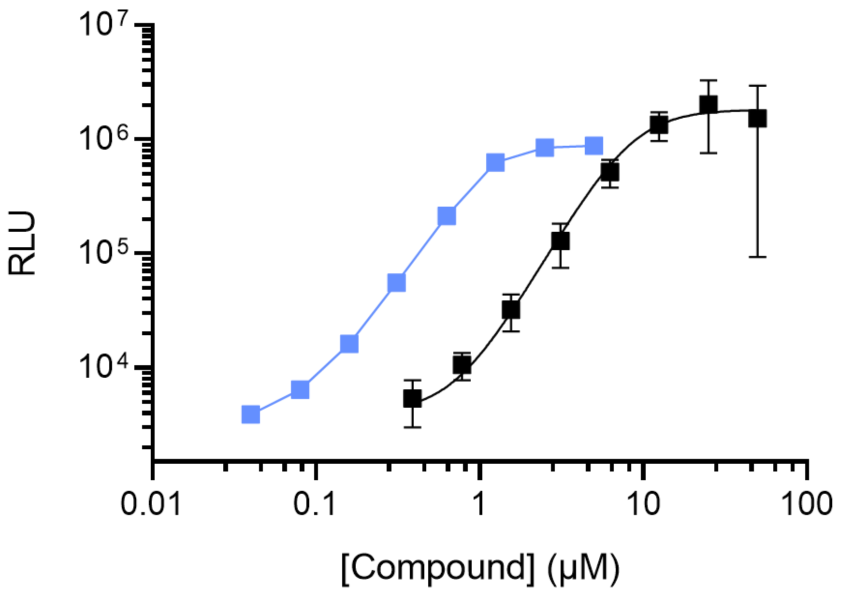

	EC50 (µM)
Compound **1**	1.0
Compound **8**	8.3

Figure S10. Light output from the *V. cholerae* AI-2 reporter in response to indicated amounts of compound **1** (blue) and **8** (black). The calculated EC50 values are shown in the table below. Error bars represent standard deviations of technical replicates, n = 3.

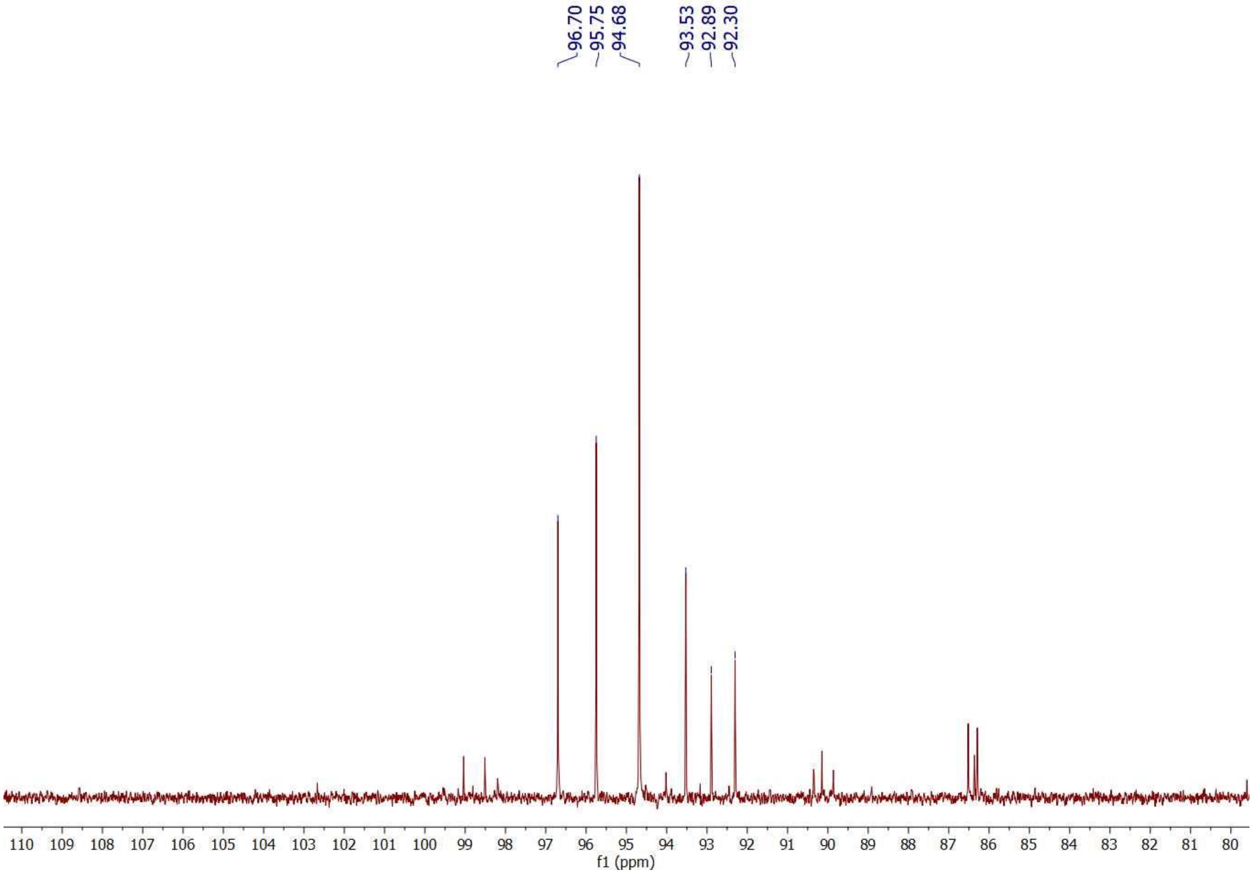

Figure S11. Expansion of the acetal region for ^{13}C NMR spectrum of compound **8**. Six major peaks are annotated with their corresponding chemical shifts in ppm. The spectrum was recorded in 95/5 H_2O/D_2O mixture.

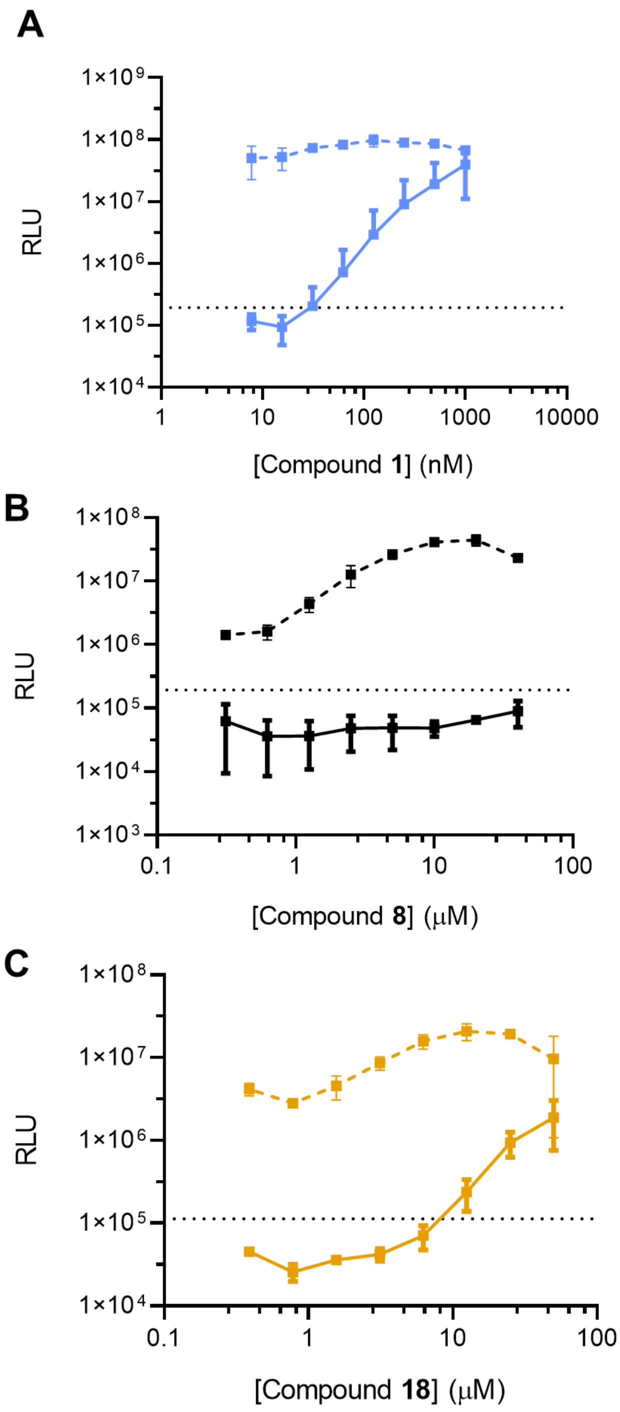

Figure S12. Bioluminescence output from the *V. harveyi* TL-26 bioassay strain in response to compounds **1** (A), **8** (B), and **18** (C). Solid and dashed lines indicate no addition or addition of 0.1 mM boric acid to the medium. Error bars denote standard deviations for biological replicates, $n = 3$.

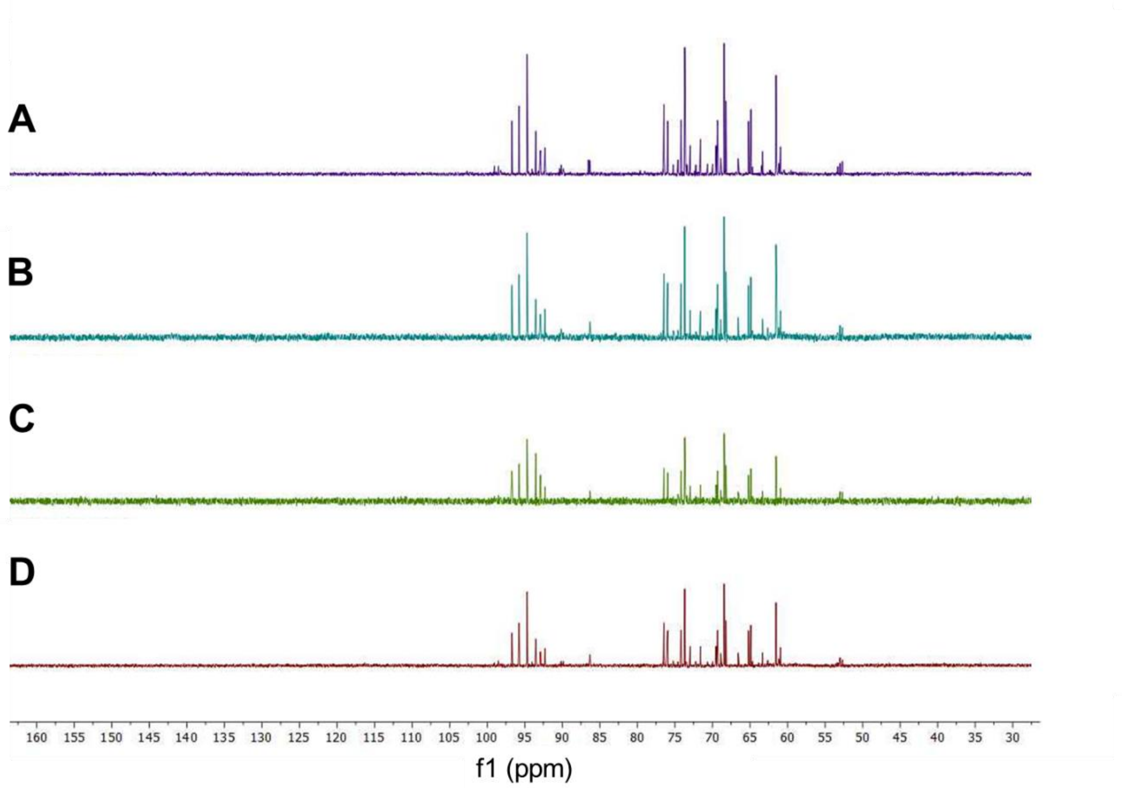

Figure S13. ^{13}C NMR comparison of 50 mM compound **8** titrated with different concentrations of boric acid. A) Compound **8** alone, B) 2:1 boric acid/compound **8**, C) 1:2 boric acid/compound **8**, D) 1:1 boric acid/**8**. All spectra were recorded in 95/5 H_2O/D_2O mixture.

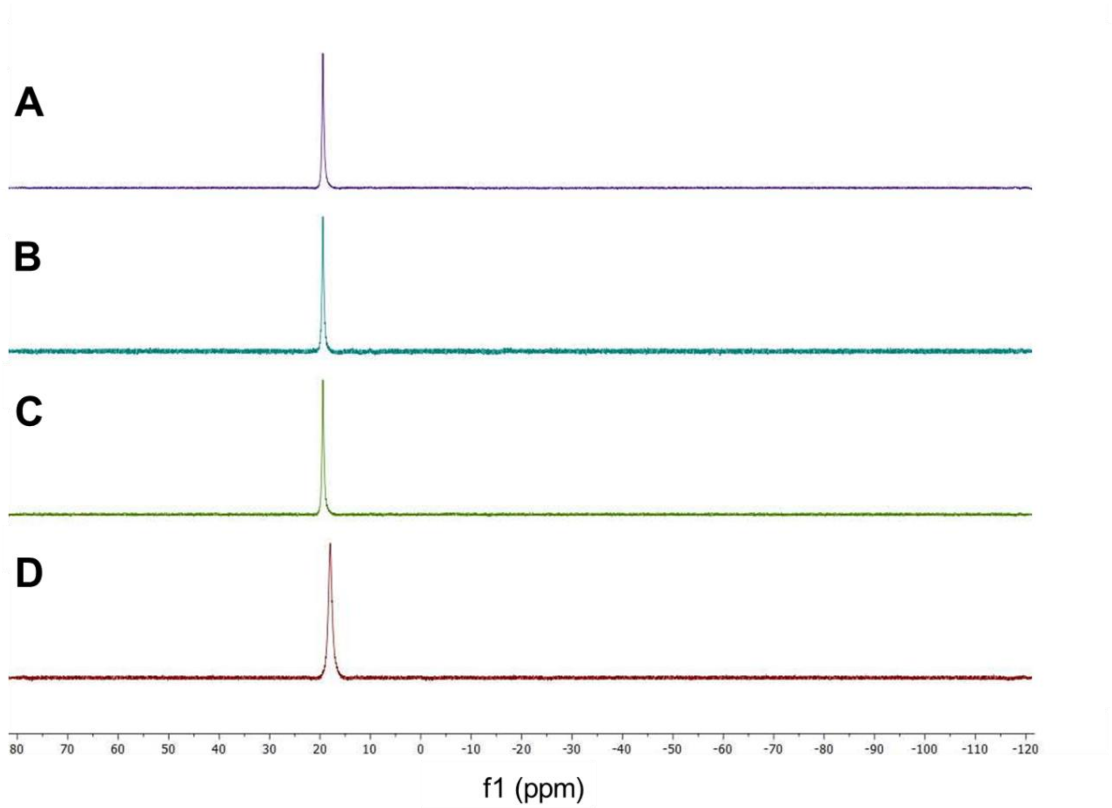

Figure S14. ^{11}B NMR comparison of 50 mM compound **8** titrated with different concentrations of boric acid. A) 2:1 boric acid/compound **8**, B) 1:2 boric acid/compound **8**, C) 1:1 boric acid/compound **8**, D) boric acid buffer standard. All spectra were recorded in 95/5 H_2O/D_2O mixture.

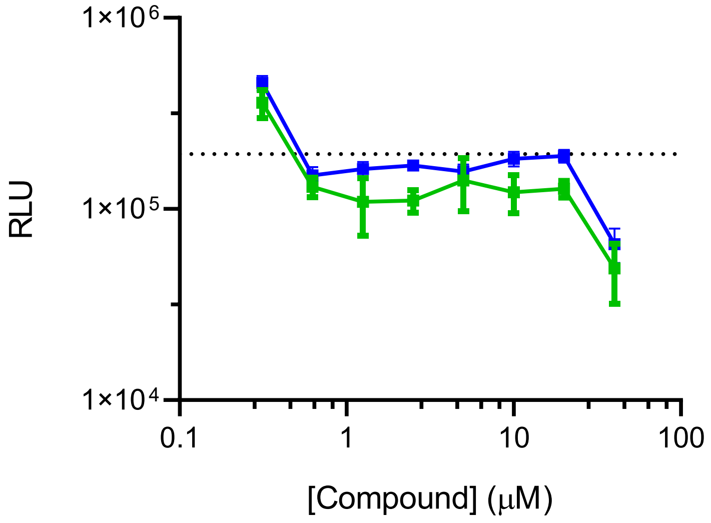

Figure S15. Bioluminescence output from the *V. harveyi* TL-26 bioassay strain in response to **10** (blue) and **16** (green). Error bars denote standard deviations for biological replicates, *n* = 3.

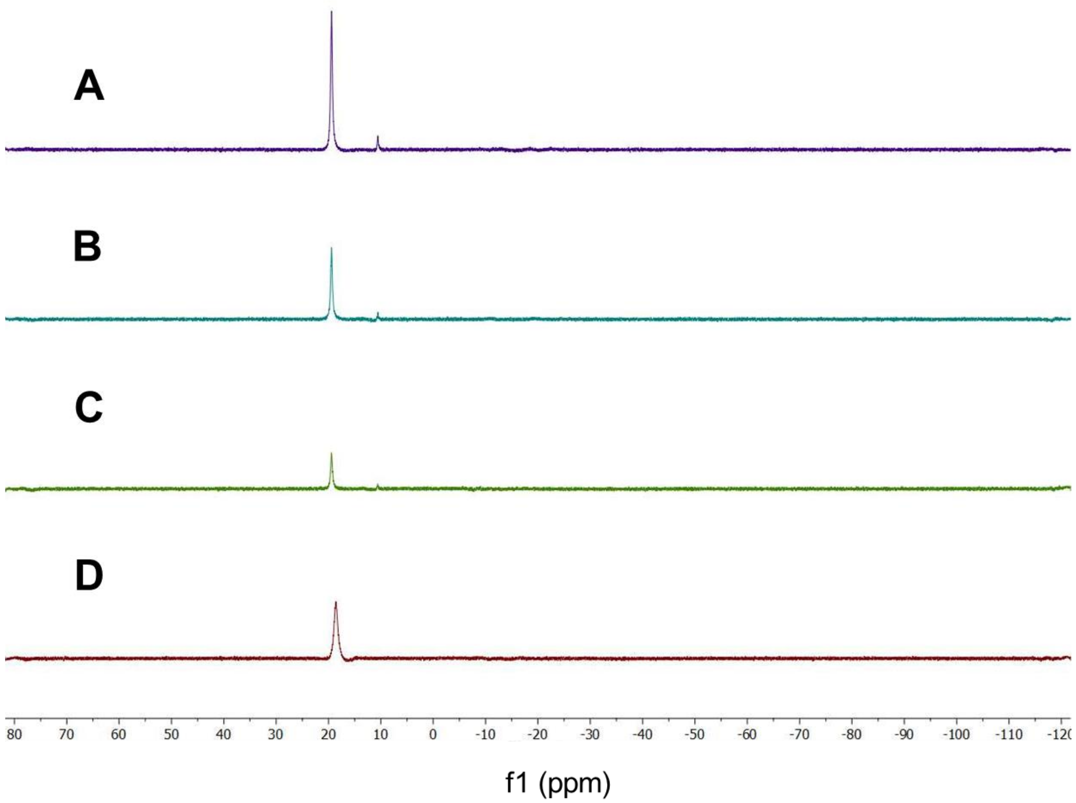

Figure S16. ^{11}B NMR comparison of 12 mM L-xylulose (compound **18**) titrated with different concentrations of boric acid. A) 2:1 boric acid/compound **18**, B) 1:1 boric acid/compound **18**, C) 1:2 boric acid/compound **18**, D) boric acid buffer standard. The peak at 19.4 ppm corresponds to borate. A small peak at 10.6 ppm is apparent when xylulose is present, consistent with the formation of a xylulose borate complex.

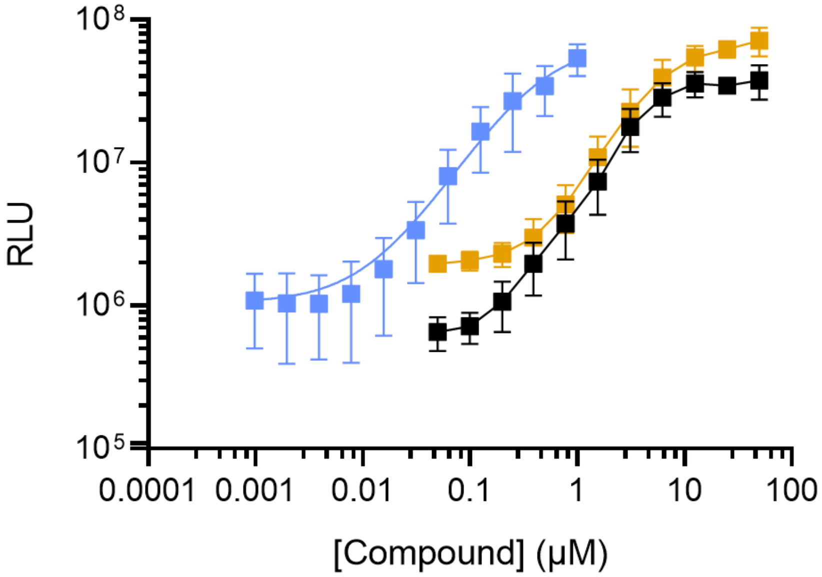

	EC50 (μM)
Compound **1**	0.4
Compound **8**	3.4
Compound **18**	6.0

Figure S17. Direct comparison of measured EC50 values of AI-2 and associated mimics in *V. harveyi* TL26 bioassay. Light output is shown in response to indicated amounts of compounds **1** (blue), **8** (black), and **18** (orange). Error bars represent standard deviations of technical replicates, n = 3.

Table S1. Significant molecular features identified in reactivity-based metabolomics workflow. Observed proton adduct [M+H], retention time (RT), raw abundance, fold change, and qvalue are shown.

m/z	RT (min)	Average Abundance (compound **5** in PBS dataset)	Average Abundance (mimic + compound **5** dataset)	Fold-Change	log2-fold	qvalue
175.0293	6.154642	1447769.385	16578944.9	11.45137	3.517448	0.005657932
176.0371	5.662175	57839.67554	1490778.225	25.77432	4.687862	0.00702287
193.0698	6.157067	15226.33997	157053.8592	10.31462	3.366618	0.005865608
205.0455	4.035233	4082.359431	841678.6448	206.1746	7.687722	0.009312385
205.0457	5.918442	107133.2131	9379938.709	87.55398	6.452101	0.008011735
206.0491	5.916925	9785.030172	975375.0917	99.68034	6.639237	0.007383072
228.9814	5.951092	2640.859709	275591.4874	104.3567	6.70538	0.001939699
235.0604	5.8959	8329.691444	700092.3232	84.04781	6.393138	0.004965719
235.0611	5.662258	43623.50743	3116981.557	71.45188	6.1589	0.009586139
236.0643	5.661342	5954.021736	384370.5411	64.55646	6.012489	0.009767693
247.0621	5.12125	8822.884968	548536.3721	62.172	5.958193	0.005239107
251.0939	6.27715	11300.7799	604859.7812	53.52372	5.742106	0.002042412
253.1211	8.300392	163869.2106	9951.560014	16.46669	-4.04148	0.005222014
254.0771	5.663183	11895.98731	872676.458	73.35889	6.1969	0.00932076
265.0749	5.5545	8156.206224	654245.6078	80.21445	6.32579	0.008802364
265.0749	5.1154	26201.47268	2552119.838	97.40368	6.605904	0.009245141
268.9739	6.957142	1206809.672	24092.07542	50.09156	-5.6465	0.001647105
283.0874	5.55685	16121.94232	3484077.422	216.1078	7.755607	0.008927702
283.1247	5.110192	32363.98125	800851.5304	24.74515	4.629074	0.001737254
296.0601	5.946483	2714.484245	391444.3905	144.2058	7.171985	0.006714692
297.104	5.776733	2287.018179	1311307.642	573.37	9.163323	0.006770262
298.107	5.747967	752.4114117	196617.0145	261.3158	8.029651	0.008540012
350.9769	6.979908	500007.7689	1566.322303	319.2241	-8.31843	0.006201043
355.1315	5.884517	1735.116785	4851601.044	2796.124	11.44921	0.004105654
363.0576	5.14435	1553.706522	1603381.713	1031.972	10.01119	0.009534706
393.0692	4.89445	274.8622079	4751215.462	17285.81	14.0773	0.004884196
434.0956	5.304133	186.3713771	1232156.327	6611.296	12.69072	0.004034585

Table S2. Top 10 most abundant fragment ions in MSMS compound **5**-derivatized AI-2 mimic. Collision energy = 20 eV.

Height	*m/z*
11969.37	175.0295
14399.21	189.0483
16267.88	206.0499
21506.45	219.0644
23697.15	247.0629
24766.35	203.0304
25215.67	201.0506
34137.32	265.0756
34171.21	176.0375
36435.18	235.0616
90029.63	205.0464

Table S3. Top 10 most abundant fragment ions in MSMS compound **5**-derivatized synthetic L-xylosone (compound **8**). Collision energy = 20 eV.

Height	*m/z*
12426.39	189.0487
16464.8	175.0299
21282.8	206.05
24707.37	219.0644
25019.65	247.0635
25350.53	201.0506
29678.37	176.0379
29910.82	203.0304
35991.71	265.0761
41043.22	235.0619
145570.7	205.0465

Synthesis of compounds ***8*** *and* ***9***

Step 1: Synthesis of Compound ***13***

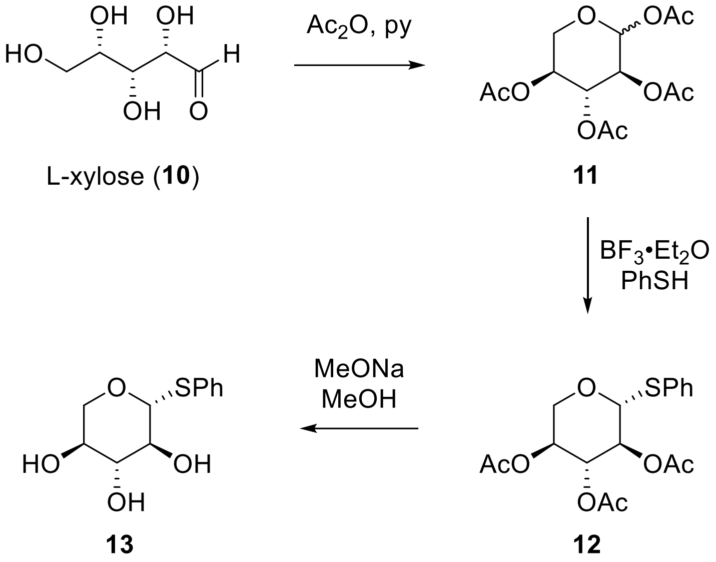

To a 250 mL round-bottom flask containing compound **10** (20 mmol, 3.00 g) was added Ac2O (200 mmol, 18.9 mL, 10 equiv) and pyridine (200 mmol, 10 equiv, 16.1 mL). The mixture was heated at 80° C for 2 h. After cooling to rt, MeOH (10 mL) was added and stirred for another 30 min. The mixture was diluted in EtOAc (200 mL), washed with 1 M HCl (200 mL), H_2O (200 mL), satd. $NaHCO_3$ (200 mL) and brine (200 mL), dried over Na_2SO_4, filtered and concentrated *in vacuo* to afford compound **11**.

To a solution of compound **11** prepared above in dry CH_2Cl_2 (30 mL) were added thiophenol (2.46 mL, 24 mmol, 1.2 equiv) and $BF_3{\cdot}Et_2O$ (7.37 mL, 60 mmol, 3.0 equiv) at 0 °C under argon atmosphere. The solution was stirred for 2 h at room temperature and then diluted with CH_2Cl_2 (20 mL). The mixture was neutralized by the addition of satd. $NaHCO_3$ (100 mL), extracted with CH_2Cl_2 (2×50 mL), dried over Na_2SO_4, filtered and concentrated *in vacuo*. The residue was dissolved in MeOH (20 mL) and MeONa (4 mmol, 1.0 M in MeOH, 4 mL) was added. After stirring for 15 min at rt, the mixture was neutralized with NH_4Cl (5 mmol, 267.5 mg) and concentrated *in vacuo*. The dried material was redissolved in Me_2CO, filtered, and concentrated *in vacuo*. After recrystallization of the residue from acetone-hexanes, compound **13** was obtained (3.49 g, 72% over 3 steps) as an off-white solid.

^{1}H-NMR (500 MHz, acetone-d_6) δ 7.50 (d, *J* = 7.6 Hz, 2H), 7.32 (t, *J* = 7.5 Hz, 2H), 7.26 (t, *J* = 7.4 Hz, 1H), 4.71 (d, *J* = 8.5 Hz, 1H), 4.38 (dd, *J* = 11.9, 4.5 Hz, 1H), 4.22 (d, *J* = 4.5 Hz, 1H), 3.98 (dd, *J* = 11.3, 4.7 Hz, 1H), 3.52 (td, *J* = 8.7, 4.5 Hz, 1H), 3.49 – 3.41 (m, 1H), 3.35 – 3.26 (m, 2H). The characterization data we obtained match those reported previously.[1]

Step 2: Acetonide protection of Compound ***13***

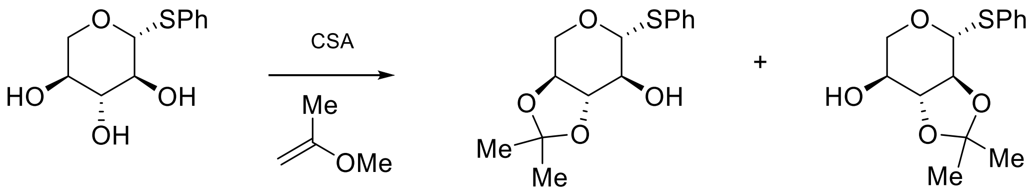

13 **14a** minor (desired regio-isomer) **14b** major (undesired regio-isomer)

To a 100 mL round bottom flask containing the triol compound **13** (2.04 g, 8.26 mmol) and (+)-camphor sulphonic acid (CSA, 192.0 mg, 0.82 mmol) in dry DMF (40 mL), 2-methoxypropene (2.38 mL, 24.8 mmol) was added dropwise under nitrogen atmosphere. The resulting mixture was stirred at rt for 2 h. Saturated sodium bicarbonate solution (160 mL) was added to the reaction mixture and stirred for another 30 min. The mixture was diluted with EtOAc (300 mL), washed with water (150 mL), brine (150 mL), dried over Na_2SO_4 and concentrated *in vacuo*. The crude product was purified on SiO_2 (Hexanes/EtOAc 10:1 to 3:1) to afford both regio-isomers of the acetonide protected sugar (major isomer: 1.78 g, 6.31 mmol, 76%, off-white solid; minor isomer: 223.2 mg 0.79 mmol, 10%, off-white solid).

Compound **14b** (major regio-isomer): ^{1}H-NMR (500 MHz, acetone-d_6) δ 7.53 (d, J = 6.5 Hz, 2H), 7.42 – 7.25 (m, 3H), 4.94 (d, J = 9.5 Hz, 1H), 4.57 (d, J = 4.8 Hz, 1H), 3.98 (dd, J = 11.5, 5.3 Hz, 1H), 3.91 – 3.79 (m, 1H), 3.59 (t, J = 9.1 Hz, 1H), 3.26 (dd, J = 11.5, 9.1 Hz, 1H), 3.18 (t, J = 9.1 Hz, 1H), 1.39 (s, 3H), 1.36 (s, 3H); ^{13}C-NMR (126 MHz, acetone-d_6) δ 133.8, 132.8, 129.7, 128.4, 110.8, 85.5, 83.7, 76.3, 71.0, 69.6, 27.0, 26.7. The characterization data we obtained match those reported previously.[2]

Compound **14a** (minor regio-isomer**):** ^{1}H-NMR (500 MHz, acetone-d_6) δ 7.54 (d, J = 7.4 Hz, 2H), 7.33 (t, J = 7.3 Hz, 2H), 7.29 (t, J = 7.2 Hz, 1H), 4.75 (d, J = 4.4 Hz, 1H), 4.59 (d, J = 8.5 Hz, 1H), 4.13 (dd, J = 9.8, 4.4 Hz, 1H), 3.65 – 3.56 (m, 2H), 3.54 (t, J = 9.1 Hz, 1H), 3.48 – 3.40 (m, 1H), 1.37 (s, 6H); ^{13}C-NMR (126 MHz, acetone-d_6) δ 134.9, 132.3, 129.7, 128.1, 111.4, 90.0, 83. 9, 74.2, 72.7, 68.5, 27.0, 26.8.

*Step 3: Synthesis of Compound **15***

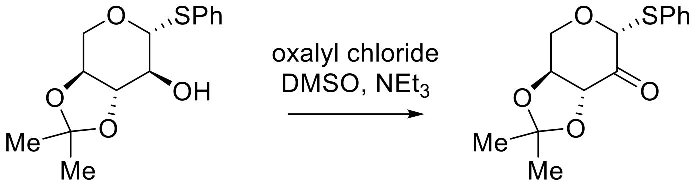

14a **15**

Oxalyl chloride (70 µL, 0.8 mmol) was dissolved in CH_2Cl_2 (4 mL) in a flame-dried 25 mL round-bottom flask and cooled to -78 °C. Dimethyl sulfoxide (DMSO, 70 µL, 1.0 mmol) was added dropwise and allowed to stir for 15 min. A solution of compound **14a** (112.9 mg, 0.4 mmol) in CH_2Cl_2 (2 mL) was added and stirred at -78 °C for 1 h followed by NEt_3 (167 µL, 1.2 mmol). The reaction mixture was stirred for another 3 h at -78°C and quenched by the addition of H_2O (10 mL). The aqueous layer was extracted with CH_2Cl_2 (3 x 10 mL), washed with brine (10 mL), dried over Na_2SO_4 and concentrated *in vacuo*. The crude product was purified on SiO_2 (Hexanes/EtOAc 10:1 to 5:1) to afford the ketone product **15** (71.8 mg, 0.26 mmol, 64% yield) as an off-white solid.

^{1}H-NMR (500 MHz, acetone-d_6) δ 7.57 (d, J = 6.6 Hz, 2H), 7.44 – 7.37 (m, 3H), 5.73 (s, 1H), 4.71 (d, J = 6.1 Hz, 1H), 4.66 – 4.57 (m, 2H), 4.05 (d, J = 13.7 Hz, 1H), 1.38 (s, 3H), 1.36 (s, 3H); ^{13}C-NMR (126 MHz, acetone-d_6) δ 200.2, 137.2, 133.3, 130.2, 129.1, 111.2, 90.2, 77.2, 76.2, 61.7, 27.3, 26.0.

*Step 4: Synthesis of L-xylosone (**8**)*

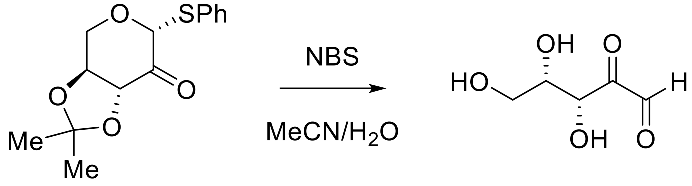

15 L-xylosone (**8**)

The protected ketone **15** (42.1 mg, 0.15 mmol) was dissolved in MeCN (1 mL) and H_2O (1 mL). N-Bromosuccinimide (NBS, 30.0 mg, 0.165 mmol) was added. The resulting mixture was stirred at rt for 10 min and quenched by the addition of NaOAc (1 mL, 1 M in H_2O). The mixture was concentrated *in vacuo* and purified on C18 modified SiO_2 (H_2O to 10% MeCN in H_2O).

The ^{1}H-NMR spectrum of xylosone appears to be complicated due to complex speciation of the sugar in water. The identity and purity of xylosone was determined by derivatization with diphenylhydrazine (DPH) shown below. DPH was chosen for this analysis because it results in a more stable product than does derivatizing with compound **5** used in the metabolomics analyses. Thus, derivatization with DPH enabled isolation and purification.

D-xylosone was prepared and analyzed using the identical sequence described above for synthesis of L-xylosone starting with D-xylose.

*Diphenylhydrazine (DPH) derivatization of compound **8***

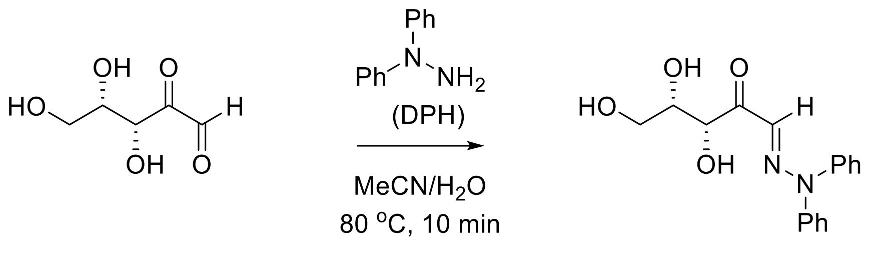

To 200 µL of the resulting fraction from the C18 purification in step 4 was added 200 µL of 0.1 M diphenyl hydrazine (DPH) solution (prepared by dissolving 1 mmol of diphenylhydrazine hydrochloride and 3 mmol of NEt_3 in 5 mL H_2O and 5 mL MeCN). The resulting mixture was reacted at rt for 24 h. The resulting mixture was purified on using HPLC to afford the DPH derivatized sugar (**19**).

^{1}H-NMR (500 MHz, MeOH-d_4) δ 7.49 (brs, 4H), 7.33 (brs, 2H), 7.22 (dd, *J* = 8.6, 1.2 Hz, 4H), 6.62 (s, 1H), 5.26 (d, *J* = 2.1 Hz, 1H), 4.29 (td, *J* = 6.7, 2.1 Hz, 1H), 3.81 (dd, *J* = 10.9, 7.0 Hz, 1H), 3.67 (dd, *J* = 10.9, 6.4 Hz, 1H);

^{13}C-NMR (126 MHz, MeOH-d_4) δ 199.2, 131.2, 75.1, 74.4, 64.0. Aromatic signals are missing from the ^{13}C-NMR spectrum due to hindered rotation.

ESI-HRMS cald. for $C_{17}H_{19}N_2O_4^+$ $[M+H]^+$ = 315.1339 m/z, found 315.1337 m/z. The characterization data we obtained match the ones reported previously.[3]

HPLC method: Kinetex 5 µm C18 100 Å, LC Column 250 x 10.0 mm (70% MeCN in H_2O, 2.5 mL/min, 15 min). DPH modified xylosone was eluted between 4.5 and 4.9 min.

Quantitation of xylosone stock solution

<u>Procedure</u>: The extinction coefficient (ε) of compound **19** was determined using HPLC with UV detection at 254 nm. A 5 mg/mL stock solution was prepared by dissolving purified compound **19** in MeCN. Serial dilutions in MeCN were made to obtain stock solutions with concentrations of 2.5, 1.25, 0.625, and 0.3125 mg/mL. Each solution was filtered through a 0.22 μm membrane before injection.

Purified compound **8** or **9** was dissolved in 10 mL water. A 400 μL aliquot of the solution was derivatized with 200 μL of 0.1 M DPH (prepared by dissolving 1 mmol of diphenylhydrazine hydrochloride and 3 mmol of NEt_3 in 5 mL H_2O and 5 mL MeCN), diluted to 1 mL using MeOH and allowed to stand for 24 h at rt before analyzing by HPLC. The concentrations of the initial xylosone solutions were calculated using the standard curve.

HPLC method: Kinetex 5 μm C18 100 Å, LC Column 250 x 10.0 mm (10 μL per injection, 70% MeCN in H_2O, 2.5 mL/min, 15 min).

^{1}H-NMR, 500 MHz, acetone-d_6

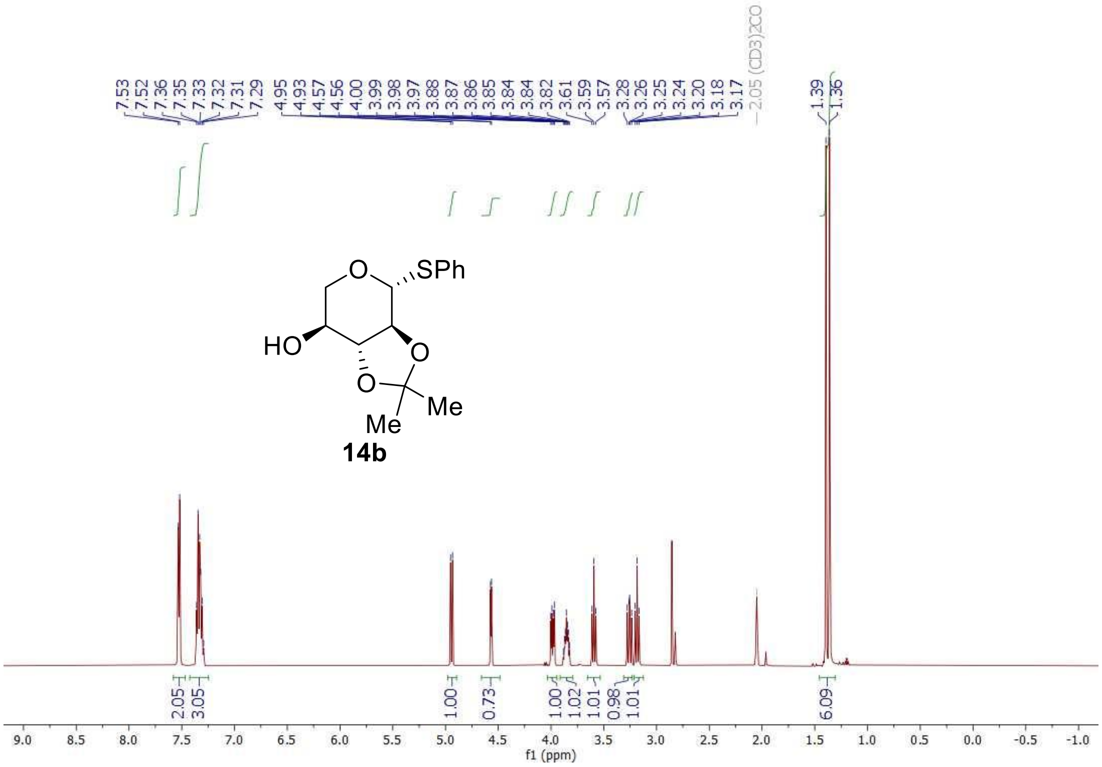

^{13}C-NMR, 126 MHz, acetone-d$_6$

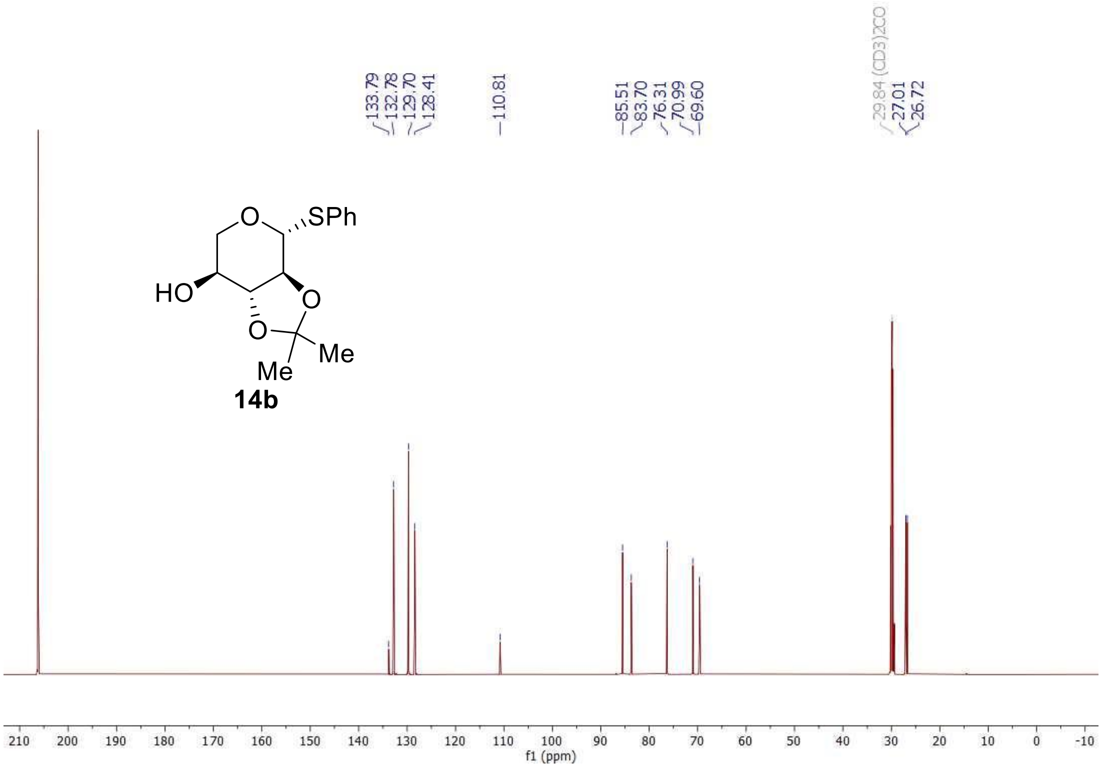

^1H-^1H COSY, 500 MHz, acetone-d_6

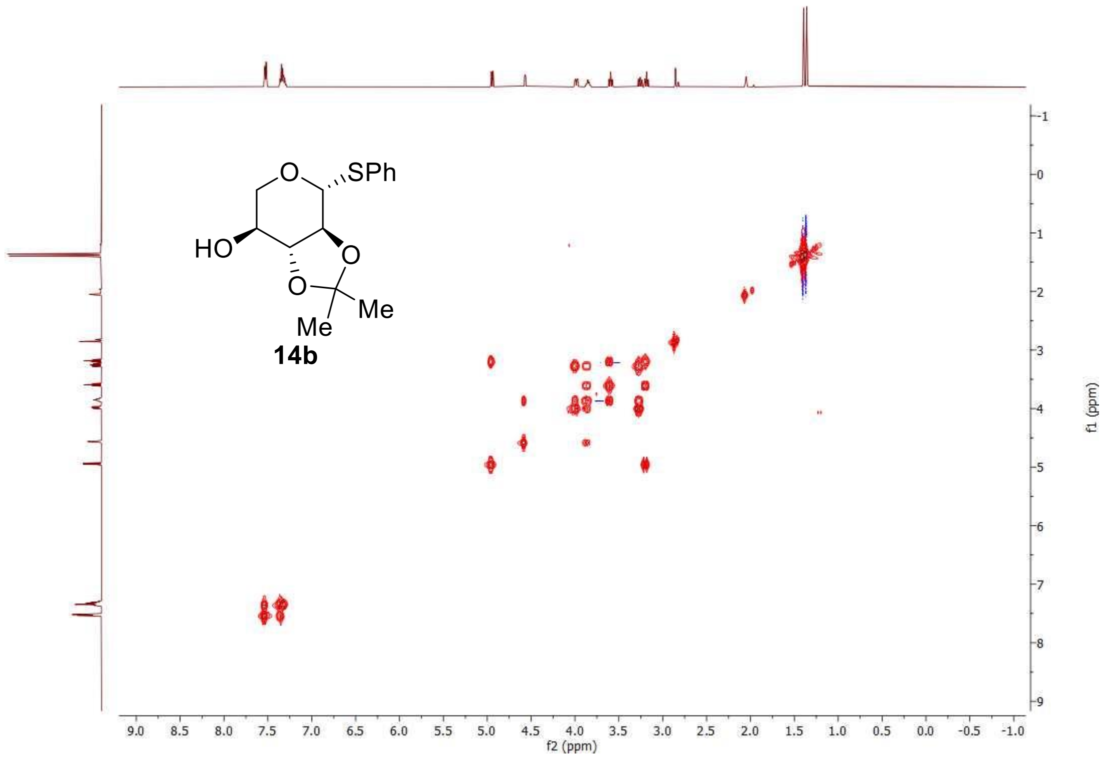

^{1}H-^{13}C HSQC, 500 MHz, acetone-d_6

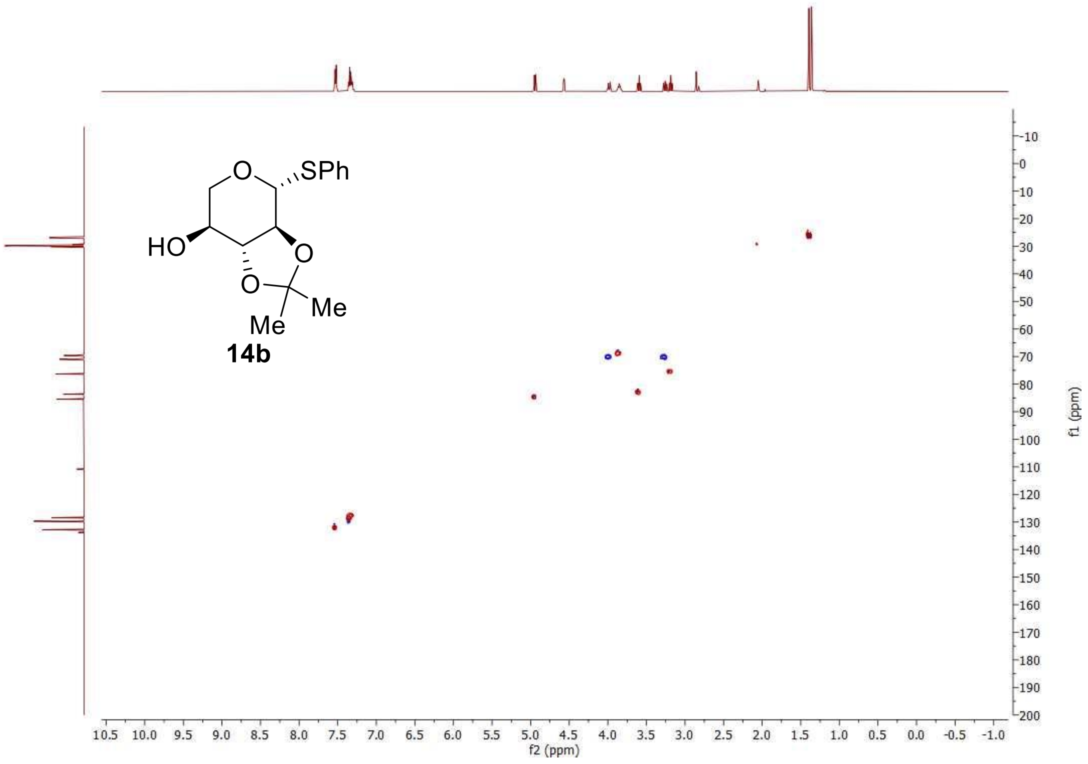

^{1}H-^{13}C HMBC, 500 MHz, acetone-d_6

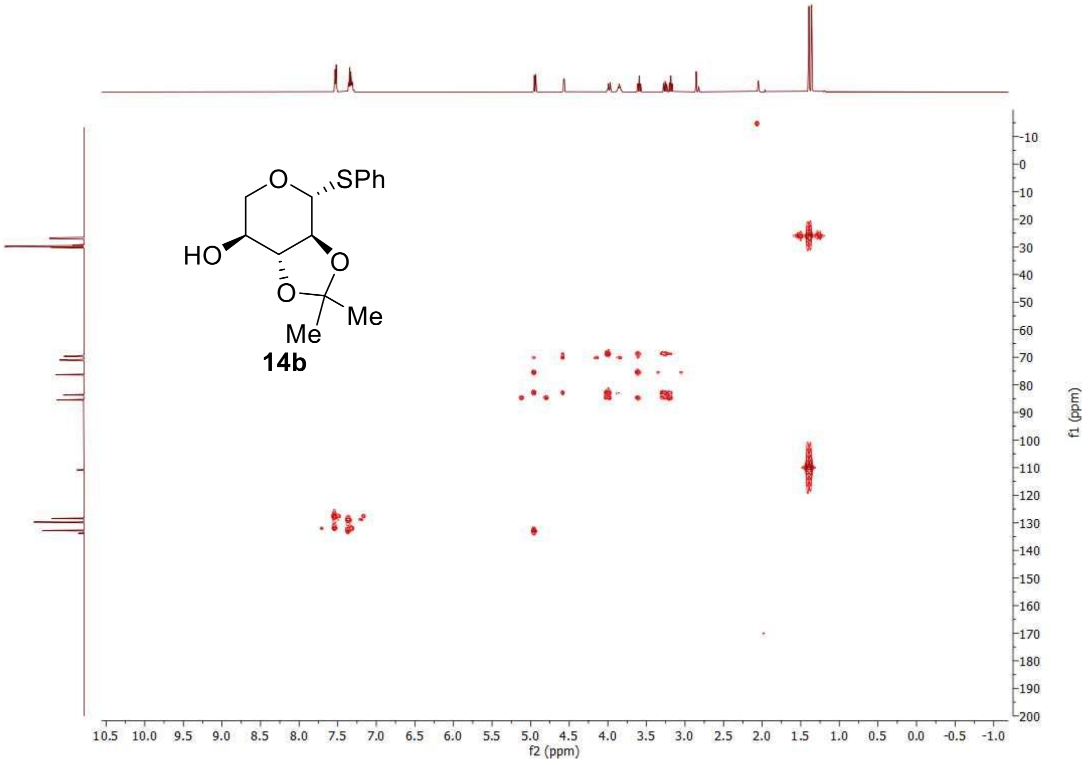

^{1}H-NMR, 500 MHz, acetone-d_6

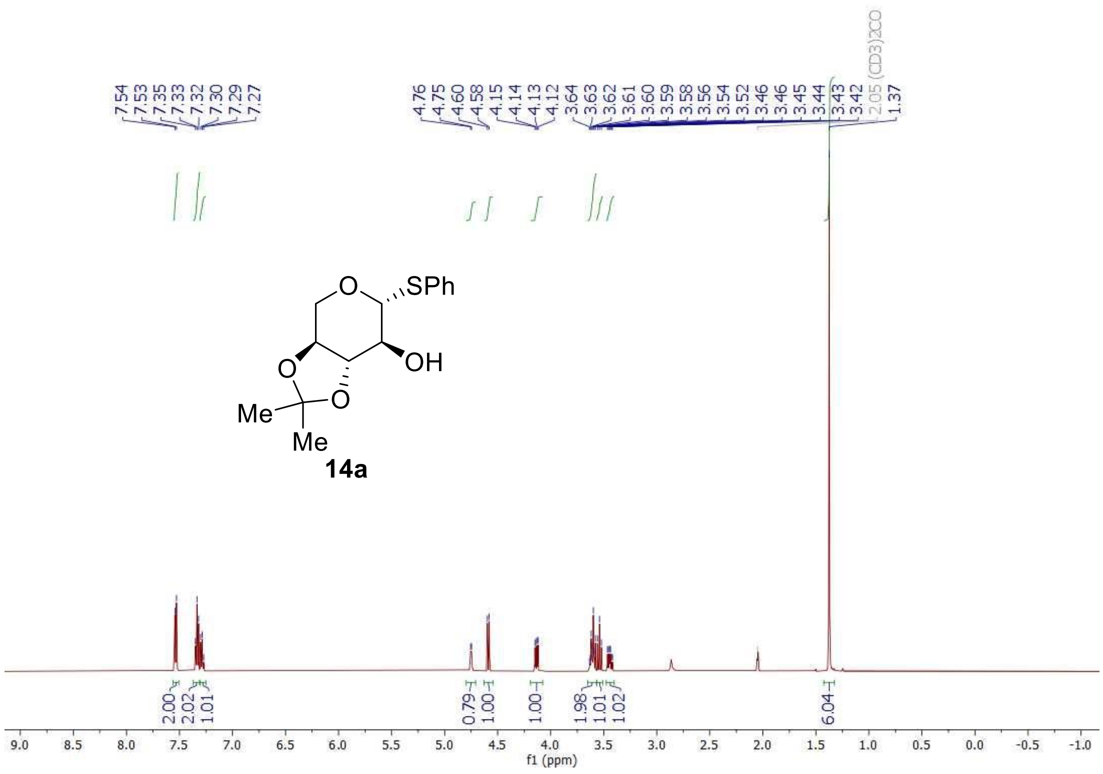

^{13}C-NMR, 126 MHz, acetone-d_6

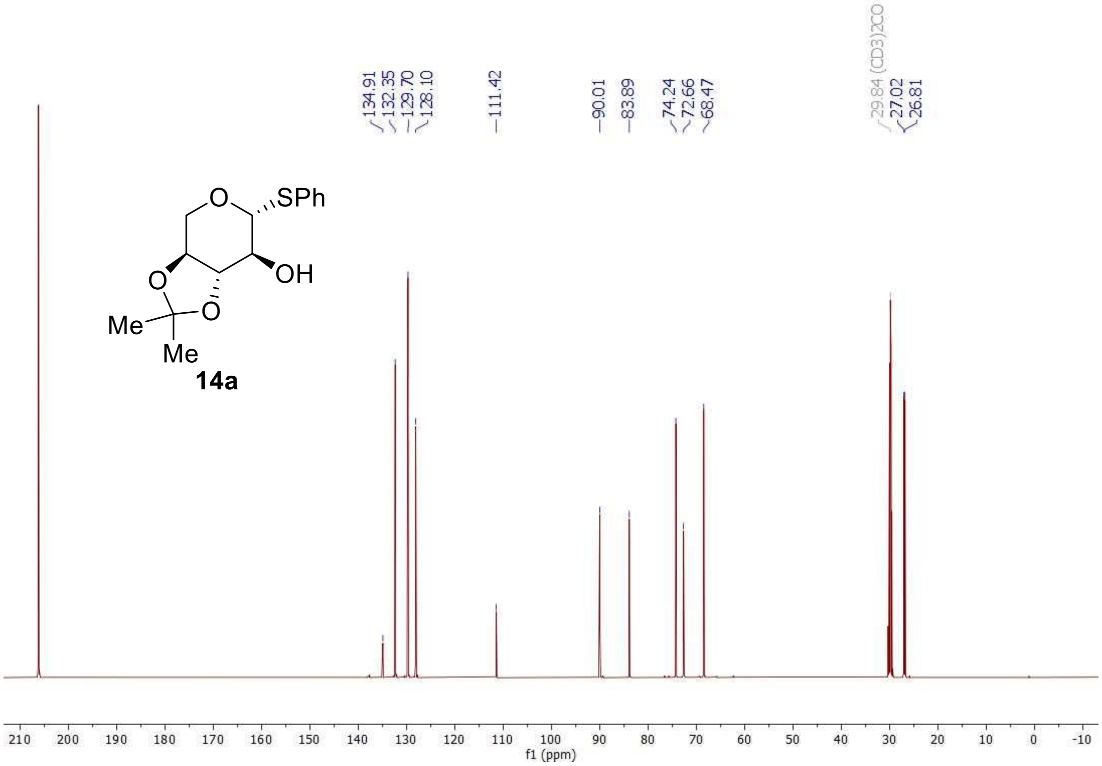

14a

^{1}H-^{1}H COSY, 500 MHz, acetone-d$_6$

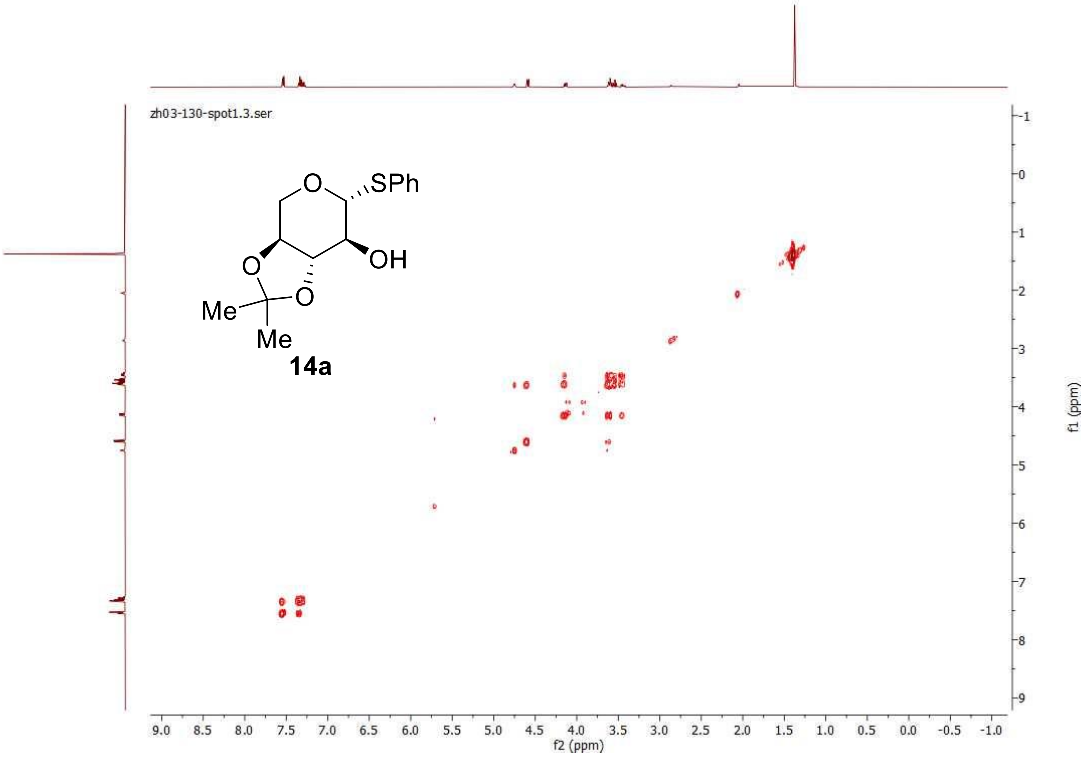

^{1}H-^{13}C HSQC, 500 MHz, acetone-d_6

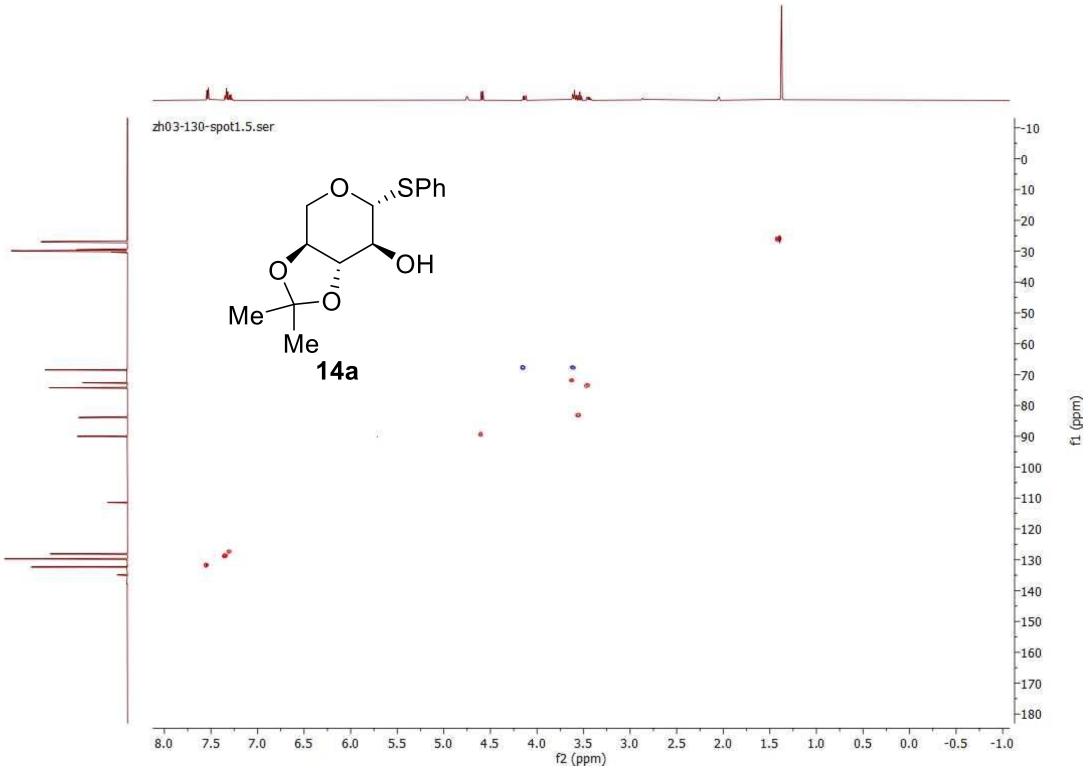

$^{1}H-^{13}C$ HMBC, 500 MHz, acetone-d_6

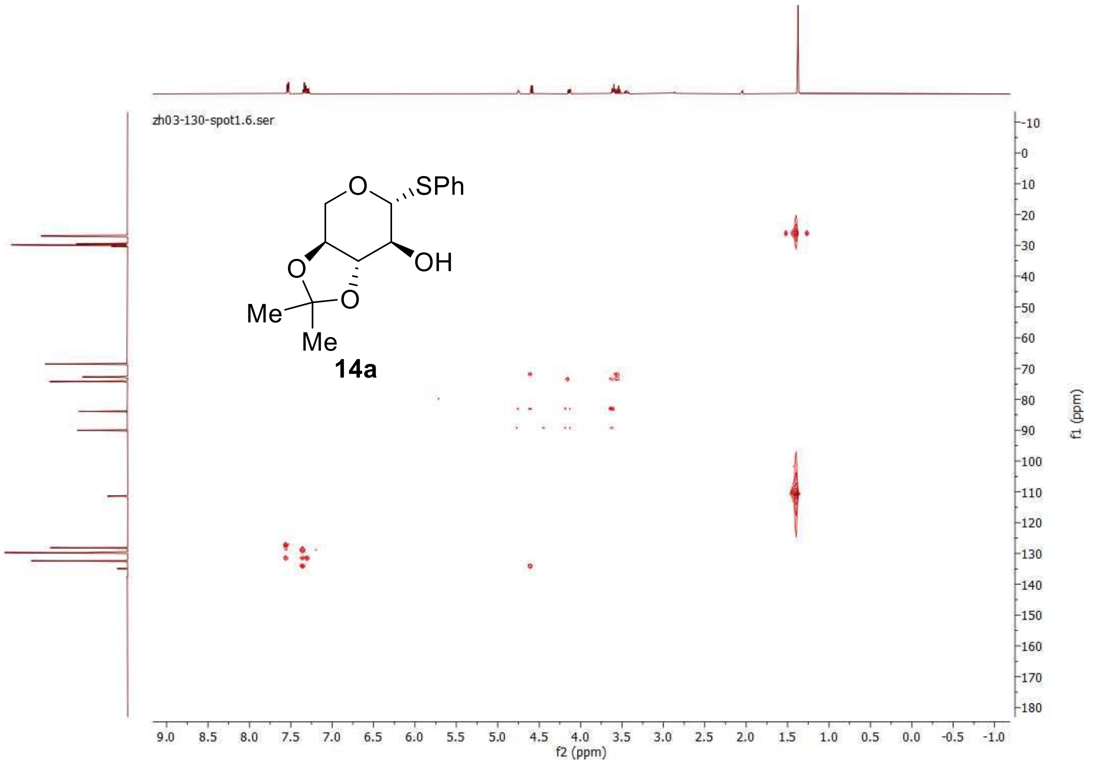

^{1}H-NMR, 500 MHz, acetone-d$_6$

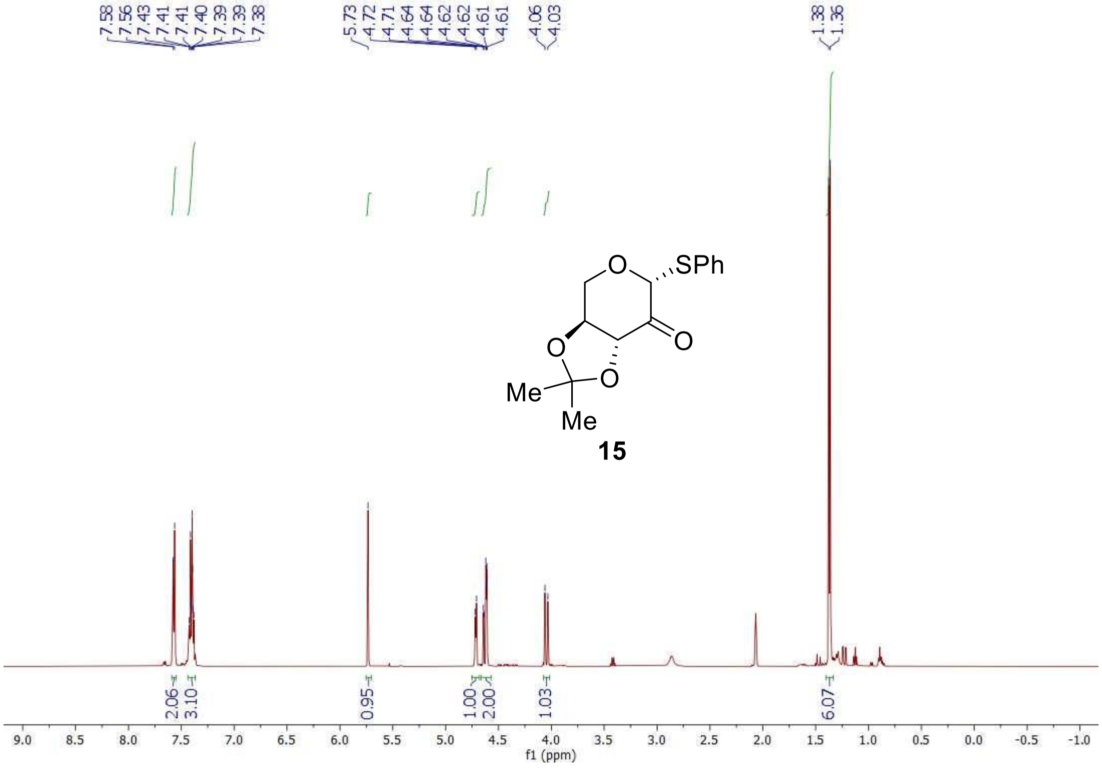

^{13}C-NMR, 126 MHz, acetone-d_6

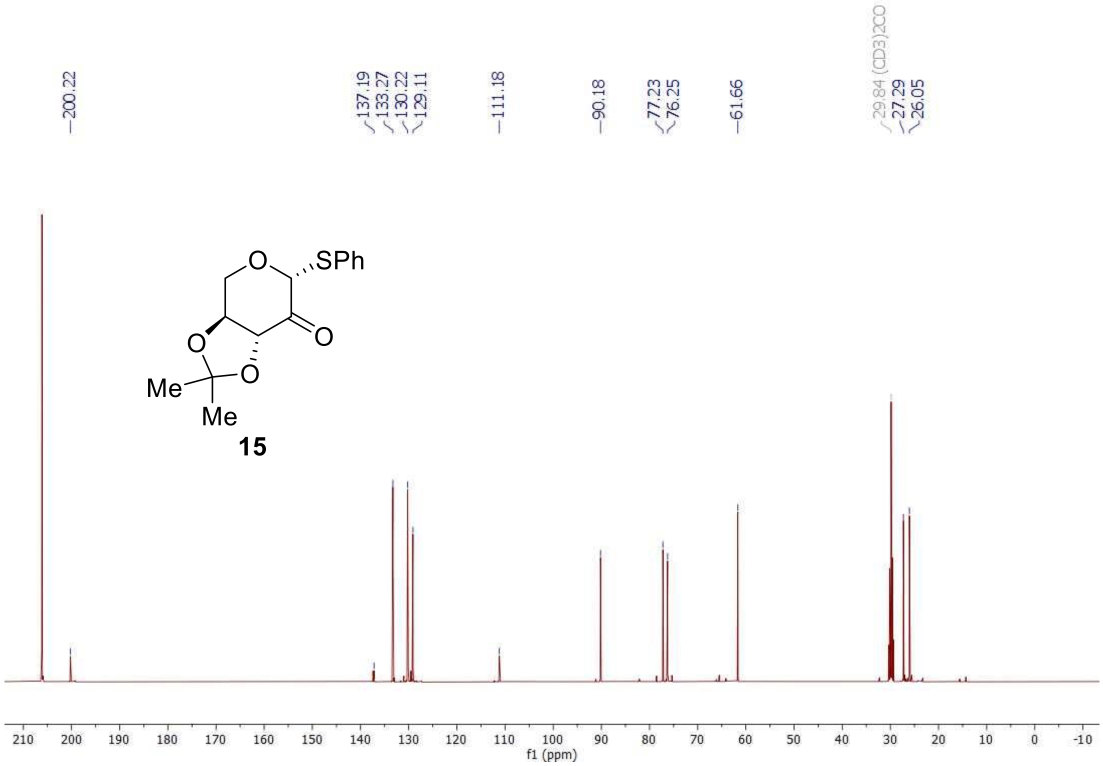

^{1}H-NMR, 500 MHz, D_2O

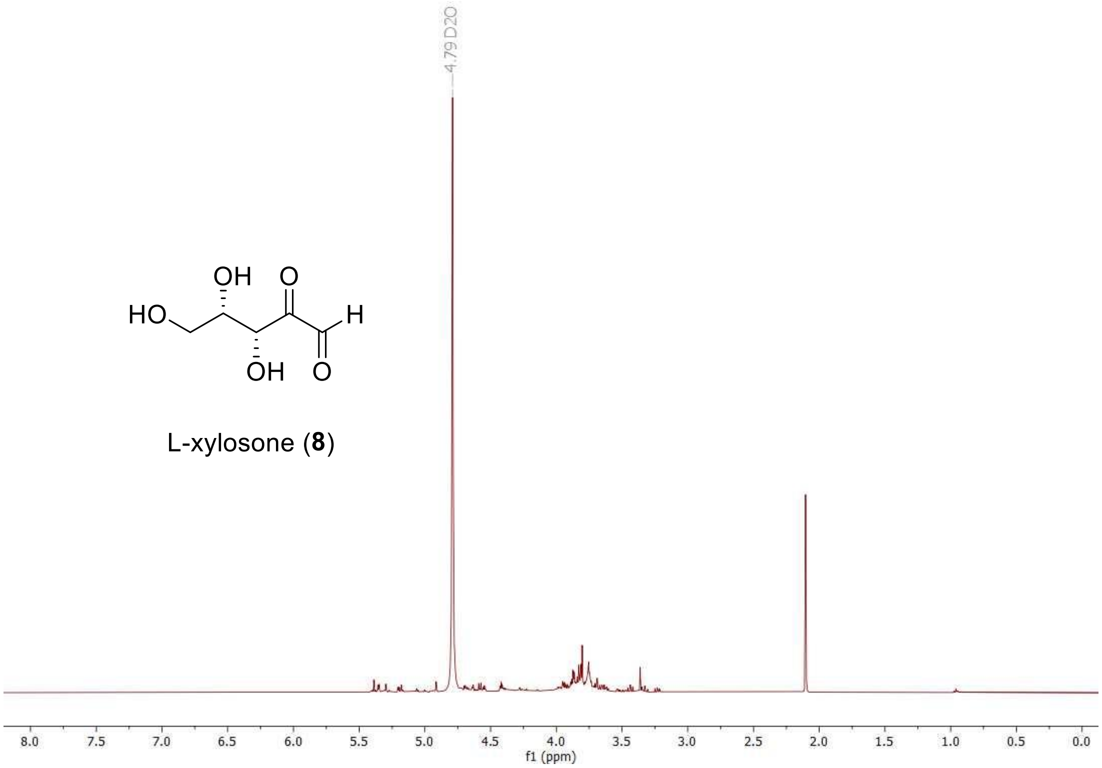

^{1}H-NMR, 500 MHz, MeOH-d_4

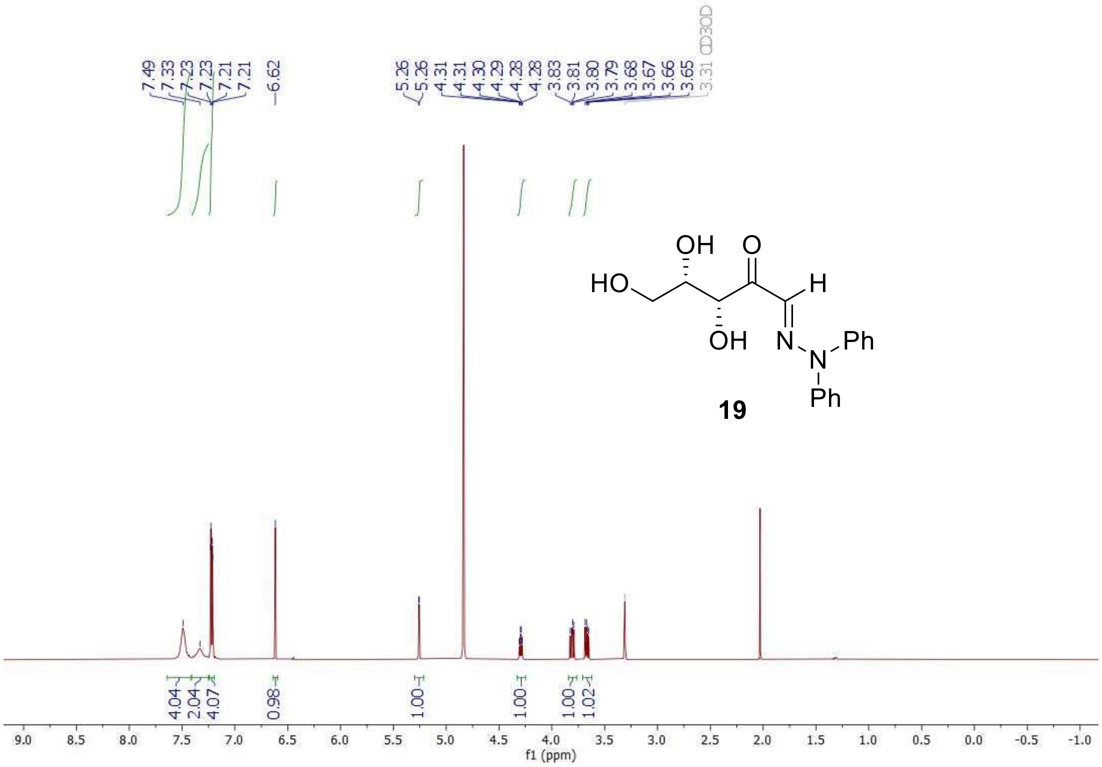

^{13}C-NMR, 126 MHz, MeOH-d$_4$

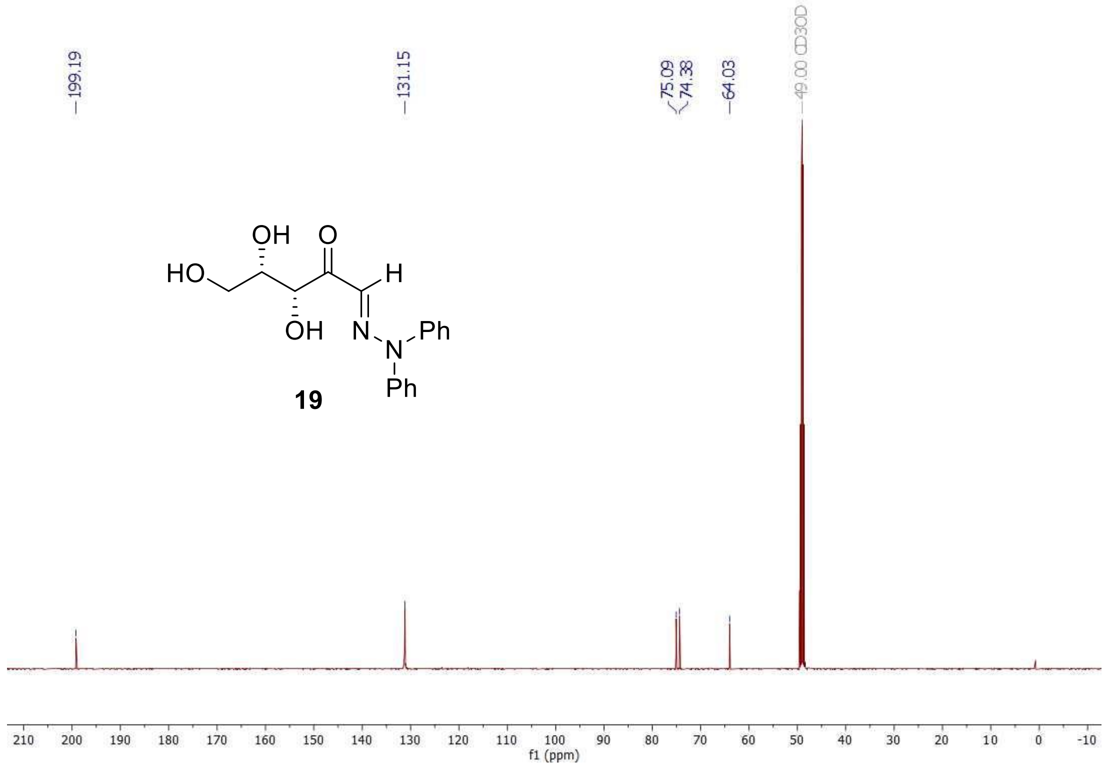

^{1}H-^{1}H COSY, 500 MHz, MeOH-d_4

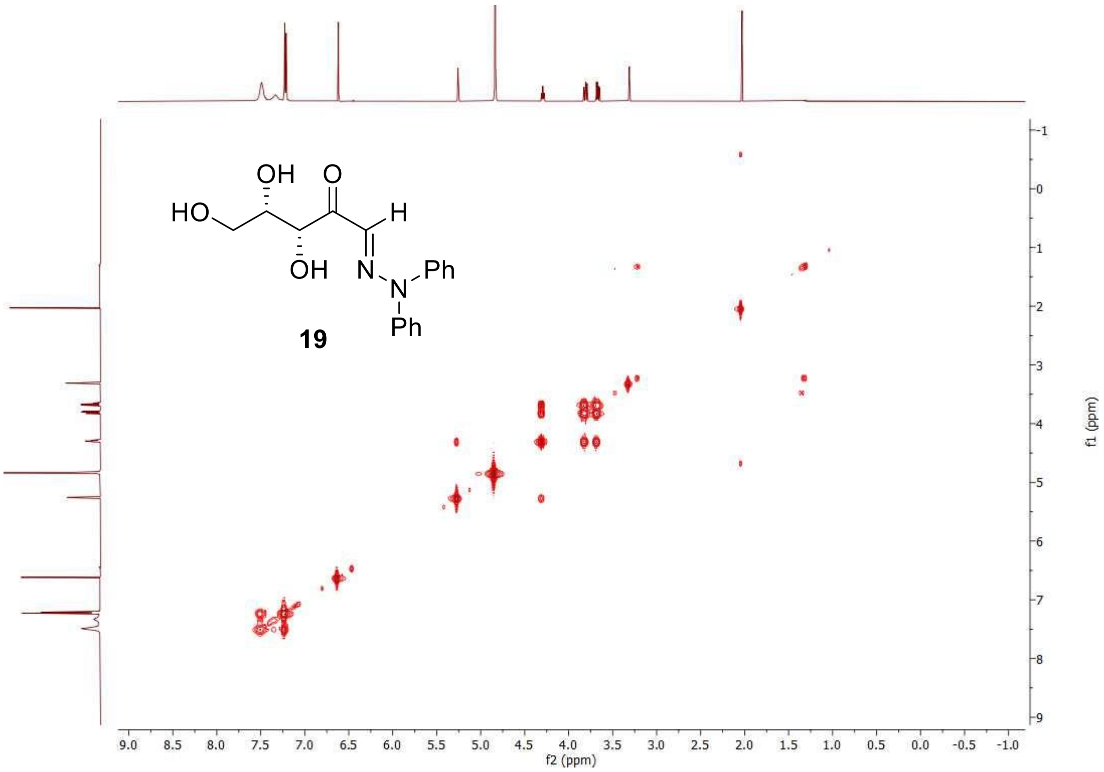

^{1}H-^{13}C HSQC, 500 MHz, MeOH-d$_4$

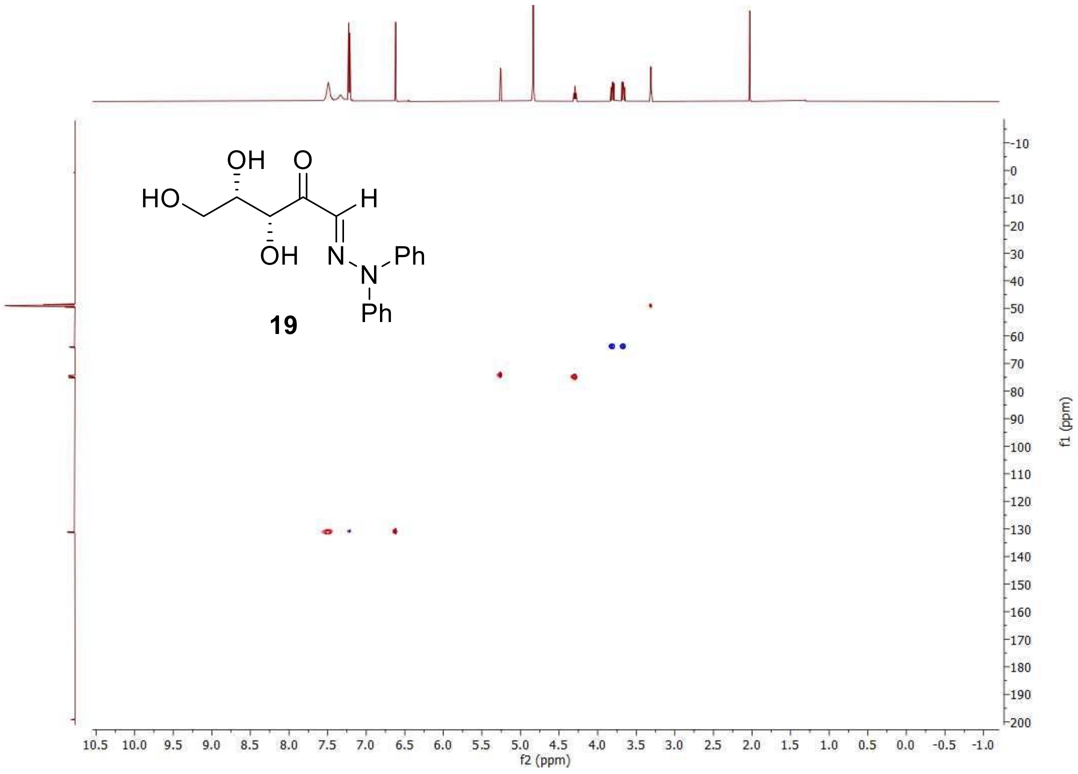

^{1}H-^{13}C HMBC, 500 MHz, MeOH-d_4

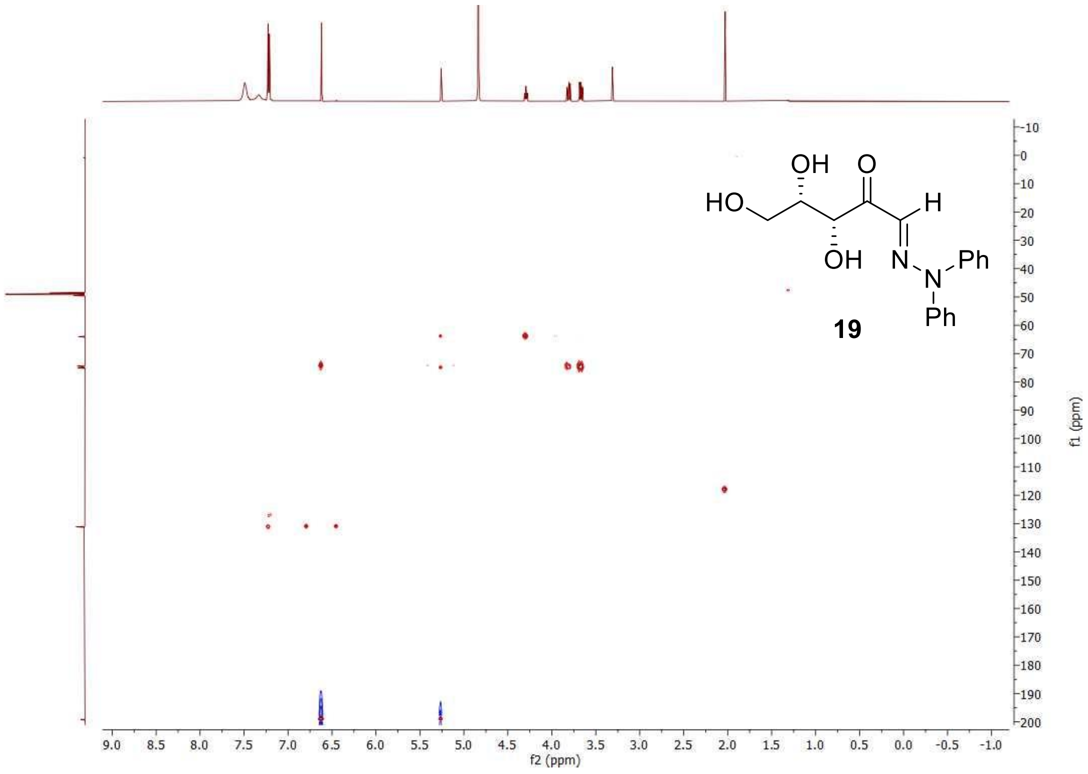

Supplementary References

1. Lopez, R. & Fernandez-Mayoralas, A. Enzymic .beta.-Galactosidation of Modified Monosaccharides: Study of the Enzyme Selectivity for the Acceptor and Its Application to the Synthesis of Disaccharides. *J. Org. Chem.* **59**, 737-745 (1994).
2. Phanumartwiwath, A., Hornsby, T. W., Jamalis, J., Bailey, C. D. & Willis, C. L. Silyl Migrations in d-Xylose Derivatives: Total Synthesis of a Marine Quinoline Alkaloid. *Org. Lett.* **15**, 5734-5737 (2013).
3. Volc, J., Sedmera, P., Halada, P., Přikrylová, V. & Haltrich, D. Double oxidation of d-xylose to d-glycero-pentos-2,3-diulose (2,3-diketo-d-xylose) by pyranose dehydrogenase from the mushroom Agaricusbisporus. *Carbohydr. Res.* **329**, 219-225 (2000).
